# Education Reform using Common Career Selective Programme (CCSP) to Promote Education-for-All (EFA)

**DOI:** 10.12688/f1000research.163862.1

**Published:** 2025-07-09

**Authors:** Frederick Agyemang, Benedicta Woode, Samuel Kofi Fosuhene, Nana Ama Browne Klutse, Alexander Kojo Nyarko, Seth Oku Asamoah, Bismark Antwi Agyei

**Affiliations:** 1 Ghana Atomic Energy Commission (GAEC), Accra, Greater Accra Region, Ghana; 2 Wisdom and Instruction Career Advisory Board (WICAB), Accra, Ghana; 3 University of Ghana (UG), Accra, Greater Accra Region, Ghana; 4 Ghana Education Service (GES), Accra, Ghana; 5 Academy for Advanced and Further Education and Training (AFAFET) Global, Accra, Ghana

**Keywords:** Education Reform, Education-for-All (EFA), Pre-Tertiary Education, Career Guidance, Common Career Selective Programme (CCSP), career and technical, Curriculum, high school, elementary school, middle school

## Abstract

Choosing a course of study in a Senior High School in Ghana is a major problem for both students and parents due to the limited number of Courses Offered. Irrespective of a student’s career aspiration, the current policies of the Ministry of Education, the Ghana Education Service, and the West Africa Examinations Council is restricted to seven main courses, namely: Agriculture, Business, Technical, Home Economics, Visual Arts, General Arts, and General Science. These courses do not reflect the numerous educational courses and career options of the world of work. Although there are schools that offers specialized programmes but the student numbers are few. Annually, hundreds of thousands of students graduate only to be confronted with another hurdle of choosing a course for further studies different from the course studied earlier, or pursue a career different from the course studied in the Senior High School. To address these challenges, this paper recommends Common Career Selective Programme, as a course of study to represent Core subjects, which consists of 18 selective courses dubbed CCSP-18. The CCSP-18 provides general course descriptions, options for specified courses, elective courses, career options, career requirements, and a list of associated careers. The CCSP-18 concept is designed to offer students variety of course options, that suite individual natural abilities and future career goals, in line with Education-for-All by the year 2030. The CCSP-18 policy brief provides policy outcomes and implications, plus actionable recommendations for education reform, and curriculum development. To improve learning outcomes for quality and efficiency, and make education relevant and accessible to all pre-tertiary students. In conclusion education reform using CCSP-18 provide options for Core Courses Offered, and career guidance to students, with basic educational knowledge to drive development agenda, and promote quality Education-for-All in Ghana, across the West Africa sub-region, Africa and other countries globally.

## Introduction

The Government of Ghana (GoG) has initiated some key education reform under the Education Strategic Plan (ESP 2018-2030) ( Amegah & Iddrisu, n.d.). The ESP is expected to contribute to the United Nations (UN) Sustainable Development Goals (SDG 4), Quality Education ( Boeren, 2019) which seeks “to ensure inclusive equitable quality education and promote lifelong learning opportunities for all”. The establishment of United Nations Educational, Scientific, and Cultural Organization (UNESCO), is aimed at building peace through education, science and culture, while International Bureau of Education (UNESCO-IBE) is to develop educational curriculums ( Jones, 2018). To this end the Incheon (South Korea) Declaration and the education 2030 framework for action is also committed to educating the community as the main driver of development. ( Adu-Gyamfi et al., 2016). Teaching and learning have evolved, hence the need for thorough education reform to make education fun, passionate, and career aspirations relevant, to fulfil the lifelong individual career goals. This policy brief paper proposes education reform to enhance lifelong learning that provides education for all.

## Education Reform

Education reform requires new policies for systematic changes of the entire structure of the education systems, specifically, in terms of courses offered and curriculums. In Ghana, the Ministry of Education (MoE) and the Ghana Education Service (GES) are responsible for policies and implementation of approved courses and programmes for the pre-tertiary education. The programmes are aimed at equitable quality formal education ( Adu-Agyem & Osei-Poku, 2012). Educational reforms also go with Standard Based Curriculum (SBC) ( Asante et al., 2024) development to attain education system for domestic and international recognition. The introduction of Technical and Vocational Education and Training (TVET) was hope to close the gap between theory and hands-on training. The TVET review to assess its effectiveness in Ethiopia proposed practical implementation based on international best practice, and policy enactment across Africa ( Hagos Baraki & van Kemenade, 2013). Currently, TVET is been implemented in many Africa countries and beyond. Education reform requires collaboration of all stakeholders. Regional organization such as the West Africa Examination Council (WAEC) ( Nweze & Obu, 2022), an examination board mandated to harmonize standardized examination, and committed to excellent education, providing quality and reliable education assessment should be part of any major education reform in the West Africa.

Education in Ghana over the years has experience education reforms ( Kuyini, 2013). The proposed new education reform requires holistic approach devoid of intermittent interference during regime change. The reforms should cover all the pre-tertiary schools: Preparatory schools’ education could mimic Montessori education, which involved the children’s natural interest. Primary Schools to provide formative learning. Junior High Schools (JHS) to provide foundation to specialized advance subjects. Senior High Schools (SHS), TVET and Science, Technology, Engineering and Mathematics (STEM) schools to provide career development programmes, as an entry point to the world of work. While tertiary education and higher educational institutes (HEI) could concentrate on Research, Development and Demonstration (RD&D) ( Sweet & Palazzi, 2015) programmes tailored to fulfil lifelong Education-for-All (EFA). The courses being offered at the SHS and the subjects is to prepare students for the world of work, which begins with the selection of Courses Offered at the SHS.

## Problem - Selection of Courses Offered

The selection of courses of study at the SHS and Senior High and Technical School (SHTS) goes a long way to influence the future career path of all students. Currently, the courses been offered in SHS/SHTS is limited to 7 main courses as shown in Table 1 (Courses Offered in SHS/SHTS).

• Over the years, the main Courses Offered have been restricted to Seven, (Reference data attached, 2021 Second Cycle Schools Register), irrespective of individual student’s career aspiration, with exception of few schools with specialized programmes.
• SHS graduates faces another challenge of choosing a course for further studies at tertiary institution, the courses offered at the university levels differ in most cases from the courses studied at the SHS.
• Most working adults and graduate students admits that the courses they offered in SHS is different from the work they do, and their career objectives.

**Table 1.** Courses Offered in SHS/SHTS.

No.	Programmes	Abbreviation	Code
1	Agriculture	AGRIC	101
2	Business	BUS	201
3	Technical	TECH	301
4	Home Economics	HOM. ECON.	401
5	Visual Arts	VIS. ARTS	402
6	General Arts	GEN. ARTS	501
7	General Science	GEN. SCI	502

The trend of limited courses offered requires education reform, to harmonize courses of study, curriculums and career orientation, for seamless transition of courses and programmes to career development in a lifelong learning and the world of work. The review of the current policy outcomes and implications calls for education reform and a new policy for the courses offered and a new curriculum.

## Methods

The method employed in the study of the courses offered and a new curriculum, use qualitative by considering the number of Courses Offered to the number of students at the high school level. Annually hundreds of thousands of students graduate from the SHS/SHTS, based on the current curriculums approved by policies of the Ministry of Education, Ghana Education Service, the West Africa Examination Council and the Government of Ghana.

## Policy Outcomes and Implications

The policy of free SHS education by Government of Ghana has seen an increase in enrollment, with the total number of Students (SHS 1), (SHS 2), and (SHS 3) per one academic year exceeding one million students. However, the main Causes Offered for these number of students still remains largely as: Agriculture, Business, Technical, Home Economics, Visual Arts, General Arts, and General Science, irrespective of individual future career ambitions.

Data tabulated from the “2021 Second Cycle Schools Register”, for the Academic Year 2019/2020. The total number of students according to the Ministry of Education and the Ghana Education Service, from Ghana Senior High Schools Annual Digest ( https://ges.gov.gh) indicate the total number of Students (SHS 1); 400,612: Total number of Students (SHS 2); 426,219: Total number of Students (SHS 3): 354,126: The grand Total number of Students: 1,180,957. The lifelong learning foundation of these millions of students per annum is routed in only 7 courses. This problem requires call-to-action for a new policy of education reform, that offers option of courses for individual career interest.

## Actionable Recommendations

Problem: Limited numbers of 7 Courses Offered:

Action: Introduction of Common Career Selective Programme (CCSP), to provide option for 18 selective programmes to represent the Core subjects as the main Course of study, in addition to other specified courses and elective courses.

Action: The CCSP dubbed (CCSP-18) policy brief provides seven points Actionable Recommendations as follows:

• Adoption and Implementation of the concept of Common Career Selective Programme (CCSP-18) by the Key Policy makers, namely the: Government of Ghana (GoG), Ministry of Education (MoE), Ghana Education Service (GES), West Africa Examination Council (WAEC), National Council for Curriculum and Assessment (NaCCA), Schools and Colleges of Teacher Training, Universities and Faculties, Conference of Heads of Basic Schools (COHBS), Conference of Heads of Assisted Secondary Schools (CHASS), Ghana National Association of Teachers (GNAT), National Association of Graduate Teachers (NAGRAT), Pre-tertiary Schools, Teachers, Students, Parents, Guardian, and the General Public.
• Funding and Development of CCSP-18 practices and structures of educational reforms which include: Occupational Standards, Curriculums development, Units Specifications, Learning Materials, Learning Outcomes, Assessment Instruments, and Marking Guidelines, Teaching Materials, Text Books, supplementary Career Guidnace, laboratories, workshops, tools, instruments, equipment, components and accessories.
• Sensitization programmes, symposium and conferences of the Policy Brief of CCSP-18 to the main stakeholders, the key students and pupil of Ghana, the students and pupil of the regional countries forming members of the West Africa Examination Council.
• Implementation of CCSP-18 model schools or a complete general rollout of CCSP-18 programmes nationwide.
• Timelines: The first batch of CCSP-18 model schools equipped with the necessary facilities, resources, and facilitators can start experimental implementation of CCSP at the beginning of 2025/2026 academic year.
• Teacher Training Colleges and Universities to begin CCSP-18 Course Base Training and Certification (CBTC), to modernize teaching and learning and enhance their knowledge and skills.
• Regional Examination Board (West Africa Examination Council) to begin work on the modalities of their assessment instruments and Certification.

Action: Once the CCSP policy is adapted, the next action is curriculum development.

Curriculum reforms globally adhered to the six-step approach to curriculum development ( Khan & Abu Salek, 2019), Step 1: Problem Identification and General Needs Assessment. Step 2: Targeted Needs Assessment. Step 3: Goals and Objectives. Step 4: Educational Strategies. Step 5: Implementation. Step 6: Evaluation and Feedback.

## Step 1: Problem Identification and General Needs Assessment

The Ghana Education Service operates 7 main courses or programmes, and two-tier subject systems, namely; 1. Core subjects, and 2. Elective subjects. The “Core subjects” represents English, Mathematics, Integrated Science, and Social Studies. These subjects are mandatory and should be taken by all students. The “Elective subjects” represents subjects that students can choose to study outside the main “Core subject”. The Electives are grouped into subject combination of learning areas and related learning areas depending on individual chosen course. The GES has also introduced common core programmes, with a standard-based curriculum aimed to develop critical thinking and problem-solving skills.

### Common Core Programme

The Common Core Programme (CCP) refer to educational initiative based on a set of academic guidelines, designed for students that prepare for Senior Secondary Certificate Examination (SSCE) and West Africa Senior Secondary Certificate Examination (WASSCE). The key features of CCP aims is to ensure that all students preparing for the examination, that are held to the same academic expectations, regardless of where they live.

The learning areas of CCP consist of the following:

1. Languages (English Language, Ghanaian Languages, French, Arabic)
2. Mathematics
3. Science
4. Creative Arts and Design (CAD)
5. Career Technology
6. Social Studies
7. Computing
8. Religious and Moral Education (RME)
9. Physical and Health Education (PHE)

These courses do not lead to any specific career choice as the targeted needs of the students, although choosing a course is career related, hence the CCP lacks the main driver of development for the pre-tertiary education.

## Step 2: Targeted Needs Assessment

The Targeted Need Assessment focuses on courses offered, and the curriculums associated with the courses for pre-tertiary students. The needs assessment recommends Common Career Selective Programme as a course of study.

### Common Career Selective Programme of Studies

The policy brief proposes Common Career Selective Programme (CCSP). The CCSP is a structured basic educational and training programme designed to help students and job seekers to explore within a broader curriculum for Senior High School, Senior High Technical School, Technical and Vocational Education and Training, and Science, Technology, Engineering and Mathematics. The CCSP concept consist of 18 set of selective courses as shown in Table 2. The CCSP curriculums provide Courses Offered, Courses Abstract, and Courses Description. CCSP curriculums covers Core subjects, Selective subjects, and Elective subjects, designed to provide basic educational knowledge of various career paths. The CCSP also provides career requirements, and career opportunities with related careers to arouse the interest of students opting for specific career education for quality EFA.

**Table 2.** Courses Offered based on CCSP-18 concept.

No	Courses Offered	Courses Abstract	Code
1	Commercial	Administration, Secretarial, and Office Support Studies	101
2	General Arts	Art, Media, Communication, and Design Studies	102
3	Agriculture Science	Farming, Fisheries, Forestry and Food Production Studies	103
4	Social Science	Humanities, Community, Civil Protection, and Social Studies	104
5	Technical / Vocational	Technical, Construction, Repairs, and Maintenance, Fashion, and Catering Studies	105
6	Engineering Science	Engineering, Innovation and Mathematics Studies	106
7	Health Science	Health Care, Medicine, and Allied Health Studies	107
8	Hospitality/Tourism	Hospitality, Hotelier, Travel and Tourism Studies	108
9	Law and Legal System	Law, Legal Systems, and Human Rights Studies	109
10	Life/Physical Science	Life, and Physical Science Studies	110
11	Management	Management, Leadership, Politics, and Authority Studies	111
12	Home Science	Home Economics, and Personal Services Studies	112
13	Industrial Technology	Industrial, Manufacturing, and Production Studies	113
14	Business and Finance	Business, Accounting, Banking, and Financial Studies	114
15	Sports and Music	Sports, Music, Acting, and Entertainment Studies	115
16	Education	Education, Training, Teaching and Learning Studies	116
17	Computer Science	Computers, Technology, IoT, and Coding Studies	117
18	Transportation	Aviation, Shipping, Railways and Vehicular Studies	118

The CCSP-18 for EFA provide an outline to develop the goals and objectives of the key stakeholders (policy makers) and the key partners (pre-tertiary students).

## Step 3: Goals and Objectives

The Goals and Objectives has two tiers under CCSP-18 for EFA: Tier-1 Stakeholders (key policy makers), and Tier-2 key partners (Pre-tertiary students).

### Goals of stakeholders - Policy makers

Provide education reform, adapt and implement policy to improve the learning outcomes (LO) to better serve the interest of the pre-tertiary students under the concept of CCSP-18 for EFA.

### Objectives stakeholders - Actionable recommendations

- Adapt and implement the policy of CCSP-18
- Provide the needed funding, resources and infrastructure
- Develop the necessary Occupational Standard
- Develop Career Core Competence
- Certification and Licensing
- Promote lifelong Learning
- Enhance problem solving skills using available tools and technology.

### Goals of Pre-tertiary students

Goals: To study the approved courses and prepare to take necessary examination and assessment for promotions and for the award of certificate.

### Objectives pre-tertiary students - Actionable recommendations

To acquire the relevant basic educational knowledge and skills captured in the various curriculums and syllables and take the necessary examination and assessment for certification and promotions:

- Preparatory Schools
- Primary Schools
- Junior High Schools
- Senior High Schools, TVET and STEM schools

The Goals and Objectives of all stakeholders require implementation as a call to action.

## Step 4: Educational Strategies

Educational strategies to review existing policies and promote new and improved policies. Here are three strategies:

• CCSP-18 for EFA require education reform and educational strategies to review existing policies and adopt quality educational reforms and facilitate teaching and learning that improve student learning outcomes. Effective strategies vary based on the subject, age group, and learning objectives.
• CCSP-18 for EFA require Active Learning that allows students to actively participate in the learning process through discussions, problem-solving, and hands-on activities to meet the diverse needs of students. Students learn by asking questions and solving problems using Technology to Integrate digital tools that enhance learning.
• CCSP-18 for EFA for Social and Emotional Learning (SEL) to focus on developing students’ emotional intelligence and interpersonal skills, and Project-Based Learning (PBL), where Students work on real-world projects over an extended period.

## Step 5: Implementation

Implementation of CCSP-18 for EFA require extensive works on the part of policy makers to enact the necessary policies and adoption. The next is the development of Unit Specifications (US), Learning Materials (LM), Learning Outcomes (LO), Assessment Instruments (Ass), Marking Guidelines (MG), Strands, and facilities and resources for all the 18 Courses and programmes for all levels of the educational hierarchy.

### CCSP-18 Courses Offered for Studies

The CCSP-18 provides the Header of the 18 selective course options, Core Course description, plus specified course description, tabulated list of careers and career requirements.

### Commercial - Administration, Secretarial and Office Support Studies

The Commercial Course offers learners basic educational knowledge in Administration, Secretarial and Office Support Studies, that equip students with the skills and experience required, to manage administrative tasks and support organizational operations effectively. This area of study is essential for the roles in business, government, education, healthcare, and other sectors.

### Administration, Secretarial, and Office Support Careers

Organizations such as industries, businesses, the private sector, and other government agencies require workers in offices and administrative sectors, to ensure the efficient and smooth running of such organizations. Table 3 shows a list of Administration, and Office Support careers.

**Table 3.** Commercial - Administration, Secretarial, and Office Support Careers.

Accounts Clerk	Data Entry Clerk	Marketing Manager
Administrative Assistant	Data Entry Operator	Medical Secretary
Administrative Coordinator	Data Entry Specialist	Office Coordinator
Administrative Manager	Database Administrator	Office Manager
Administrative Officer	Executive Assistant	Payroll Clerk
Administrator	File Clerk	Personal Assistant
Auditor	Financial Analyst	Postal Service Clerk
Bank Teller	Financial Manager	Project Administrator
Banker	Front Desk Clerk	Receiving Clerk
Bilingual Secretaries	General Office Clerk	Receptionist
Billing Clerk	Human Resources Assistant	Records Manager
Bookkeeping Clerk	Human Resources Coordinator	Scheduling Coordinator
Brokerage Clerk	Human Resources Manager	Secretary
Budget Analyst	Legal Secretary	Shipping Clerk
Cashier	Mail Carrier	Telephone Operator
Client Relations Specialist	Mail Clerk	Timekeeping Clerk
Customer Service Representative	Market Research Analyst	Typist

### Requirements: Administration, Secretarial and Office Support Careers

Most administrative and office support workers have degree from universities, or Diploma from higher school education. Other employers prefer college, vocational or secretarial and high schools job seekers.

### General Arts - Art, Media, Communication, and Design Studies

The General Arts - Art, Media, Communication, and Design Studies, provides basic educational knowledge that covers a diverse range of disciplines focused on creative, analysis, and interpretation of audio visual and media content. The Art studies involve exploration of Visual Arts, Performing Arts and Literary Arts. It focuses on both practical skills and creativity which require theoretical and practical tutelage. Media studies examine production, distribution, and consumption of media content, such as film, television, radio, and online platforms. Communication studies delve into the ways people share information through various channels, including journalism, and digital communication. Design studies focus on the principles and practices of creativity, fashion, graphic design, industrial design, and interior design.

### Art, Media, Communication, and Design Careers

Occupations in the areas of art, media, communications, and design, express their thought, feeling, ideas and current affairs in their work, provide beauty to the lives of their customers. Others express their ideas through information processing. An option of careers in Art, Media, Communication, and Design has been provided in Table 4.

**Table 4.** General Arts - Art, Media, Communication, and Design Careers.

Anchorman	Creative Director	Music Director
Anchorwoman	Curator	Musician
Animator	Dancer	Musicologist
Art Director	Design Director	Photographer
Art Therapist	Designer	Photojournalist
Artist	Digital Media Specialist	Podcast Producer
Audio Engineer	Disc Jockey (DJ)	Production Designer
Author	Editor	Proofreader
Blogger	Event Planner	Public Relations Officer
Brand Identity Developer	Fashion Designer	Public Relations Specialist
Broadcast News Analyst	Film Director	Radio Announcer
Broadcast Technician	Fine Artist	Reporter
Broadcaster	Floral Designer	Set Designer
Calligrapher	Game Designer	Social Media Manager
Camera operator	Graphic Designer	Sound Designer
Cartoonist	Illustrator	Technical Writer
Cinematographer	Industrial Designer	Television Anchor
Colorist	Interior Designer	Television Producer
Columnist	Jewelry Designer	User Experience (UX) Designer
Commercial Artist	Journalist	Video Editor
Communications Manager	Lighting Technician	Videographer
Composer	Makeup Artist	Visual Effects (VFX) Artist
Concept Artist	Media Buyer	Voice-over Artist
Content Strategist	Media Planner	Web Designer
Copywriter	Motion Graphics Designer	Writer
Costume Attendant	Multimedia Artist	

### Requirements: Art, Media, Communication, and Design Careers

A greater number of artists have natural artistic abilities. Some artists have to undergo training, while some have no formal training. However, others have a degree in fine arts from a university, college, or school of art. Most designers have diploma from polytechnics. In the field of communication, a bachelor’s degree or master’s degree is a requirement.

### Agricultural Science - Farming, Fisheries, Forestry and Food Production Studies

Agricultural Science - Farming, Fisheries, Forestry and Food Production Studies, focus on basic educational knowledge of scientific principles and best agriculture practices, including traditional means of Farming, Forestry, Fishing. Agricultural Science include related food production, storage, marketing, resource management, and environmental sustainability. The study provides basics knowledge to integrates Crop Science, Animal Science, Soil Science, Agricultural Economics, Horticulture, Agricultural Engineering and Food Science.

### Agricultural Science Careers

Agricultural science workers help to provide one of the main basic human needs, which is food. They work to produce all the food that we eat, and also some produce the materials that are used to make clothes, which also form part of the main basic needs of mankind. Careers in the Agricultural Science is listed in Table 5.

**Table 5.** Agriculture Science - Farming, Fisheries, Forestry and Food Production Careers.

Agricultural and Food Science Engineer	Biomass Plant Technician	Irrigation Specialist
Agricultural and Food Science Technician	Botanist	Landscape Agronomist
Agricultural Crop Farm Manager	Faller	Logging Worker
Agricultural Engineer	Farm Worker	Nursery Manager
Agricultural Equipment Operator	Farmer	Organic Farming Specialist
Agricultural Extension Officer	Farmers and Rancher	Pest Control Advisor
Agricultural Extension Officer and Inspector	Fish and Game Warden	Plant Pathologist
Agricultural Inspector	Fish Farmer	Precision Agriculture Specialist
Agricultural Scientist	Fisheries Biologist	Rangeland Manager
Agricultural Technician	Fisheries Manager	Silviculturist
Agriculturist	Fishing Crew	Soil and Water Conservationist
Agronomist	Floriculturist	Soil Scientist
Animal Breeder	Forest Conservationist	Sustainable Agriculture Consultant
Animal Farm Scientist	Forest Engineer	Timber Harvester
Animal Farmworker	Forest Ranger	Tree Surgeon (Arborist)
Beekeeper	Forest Technician	Veterinary Technician (Agriculture)
Biofuels Processing Technician	Forester	Wildlife Biologist (Agriculture and Forestry)
Dairy Farm Manager	Greenhouse Manager	Wood Technologist
Environmental Consultant (Agriculture)	Horticulturist	

### Requirements: Agriculture Science Careers

Agricultural Extension Officers have degrees or diplomas from Agricultural Colleges or a bachelor’s degree from a university. Mechanization and irrigation produced food on a larger scale. In the rural areas, most farmers and fishermen do not have formal education.

### Social Science - Humanities, Community, Civil Protection, and Social Studies

The Social Science - Humanities, Community, Civil Protection, and Social Studies, provide basic educational knowledge of societal structures, the role of community engagement, and strategies for ensuring public safety and well-being. The study is essential for understanding and improving the quality of life in various communities through effective governance, social support systems, emergency management, Police, Military, and Para-Military Services for defense and protection.

### Humanities, Community, Civil Protection, and Social Services Careers

People engaged in community and social services provide assistance to society, in order to improve the quality of life in their neighborhood and in the societies in which they find themselves. People depend on these workers to help them individually or as a group. They also help families solve their problems. Counselors in particular help people identify their problem and find solutions. Other social workers offer assistance in emergency cases. Careers in Community, Civil Protection, and Social Services has been provided in Table 6.

**Table 6.** Social Science – Humanities, Community, Civil Protection, and Social Services Careers.

Air Force Personnel		Police Chaplain
Artillery Personnel	Family and Youth Services Worker	Police Detective
Bailiff	Fire Fighter	Police Officer
Border Patrol Agent	Fire Inspector	Prison Chaplain
Case Manager	Fire Inspector and Investigator	Prison Officer
Child and Family Social Worker	Forensic Social Worker	Probation Officer
Child Protection Officer	Forest Fire Fighter	Public Defender (Social Justice Law)
Child Welfare Specialist	Foster Care Caseworker	Public Health Advocate
Clergy	Government Social Policy Analyst	Public Safety Officer
Community Development Officer	Homeless Shelter Coordinator	Rehabilitation Counselor
Community Health Worker	Human Rights Advocate	Search and Rescue Technician
Community Outreach Coordinator	Human Rights Lawyer	Security Consultant (Community Safety)
Community Service Manager	Human Services Administrator	Sexual Assault Crisis Counselor
Correctional Counselor	Infantry Soldier	Shelter Worker (Domestic Violence, Homeless)
Correctional Treatment Specialist	Juvenile Caseworker	Social Policy Analyst
Counselor (Substance Abuse, Mental Health, Family)	Juvenile Probation Officer	Social Services Coordinator
Crime Scene Investigator (CSI)	Librarian	Social Worker
Criminal Justice Advocate	Marriage and Family Therapist	Suicide Prevention Counselor
Curator	Mediator	Support Group Facilitator
Custom Officer	Mental Health Advocate	Victim Advocate
Detective & Criminal Investigator	Military Personnel	Violence Prevention Specialist
Disaster Response Coordinator	Municipal Fire Fighter	Volunteer Coordinator (Community Services)
Domestic Violence Counselor	Nonprofit Program Coordinator	Youth Caseworker
Drug and Alcohol Counselor	Paralegal (Community Legal Services)	Youth Counselor
Emergency Management Director	Parole Officer	Youth Development Specialist

### Requirements: Humanities, Community, Civil Protection, and Social Services

At least a High School level of education is the minimum requirement for all branches of the forces such as the Army, Air-force, Police, Fire, Prison, Customs, and Immigration services and other voluntary services. A diploma or university degree may be preferred in some cases or a professional qualification. Most members of the Clergy attend seminaries, other field within the community and social services require degree from a university.

### Technical / Vocational - Technical, Construction, Repairs, and Maintenance, Fashion and Catering Studies

The Technical / Vocational - Technical, Construction, Repairs, and Maintenance Fashion and Catering Studies, provide basic educational knowledge that focuses on basics of design, building, fitting, mechanics, technicians and other specialized sectors such as Fashion, Clothing, and Catering Services. For effective working of automobiles, electricals, electronics, structures, infrastructure, and systems, these workforces are inevitable. These fields are essential for creating and maintaining the built environment, ensuring safety and sustainability. Construction, Maintenance, Repairs and Technical skills is prerequisite as Interdisciplinary Civil Engineering, Architecture, Environmental Engineering and Urban Planning and many others, to ensure that infrastructure meets current and future needs.

### Careers in Technical, Construction, Maintenance, and Repairs

Most workers in the field of construction help in the building of houses, roads, bridges, culverts, shops and other commercial building. Maintenance and repair workers help to maintain machinery and other properties and fix them when they are faulty or not working properly. Construction, Maintenance, and Repairs careers shown in Table 7 offers option in this category.

**Table 7.** Technical / Vocational - Technical, Construction, Repairs and Maintenance, Fashion and Catering Careers.

Assembler and Fabricator	Roofer	Insulation Installer
Automatic Teller Machine Attendant	Tile and Marble Setter	Landscaper & Groundskeeper
Automotive Body Repairer	Air Conditioning Technician	Machinery Maintenance Worker
Automotive Engineer	Automatic Teller Machine (ATM) Attendant	Marine Mechanic
Automotive Engineering Technician	Boilermaker	Mason (Brick, Stone, Cement)
Automotive Glass Installer & Repairer	Bridge Construction Worker	Metal Fabricator
Automotive Master Mechanic	Cabinetmaker	Millwright
Automotive Mechanic	Carpet Installer	Oil and Gas Driller
Automotive Specialty Technician	Ceiling Tile Installer	Pipefitter
Automotive Technician and Mechanic	Cement Mason and Concrete Finisher	Plasterer
Auxiliary Power Equipment Operator	Construction Equipment Operator	Power Plant Maintenance Technician
Aviation Inspector	Construction Foreman	Railroad Maintenance Technician
Avionics Technician	Construction Laborer	Refrigeration Mechanic
Bricklayer	Crane Operator	Rigger
Carpenter	Demolition Worker	Scaffolder
Carper Installer	Diesel Mechanic	Sheet Metal Worker
Concrete Mason	Dock and Port Maintenance Technician	Shipbuilder and Repair Technician
Electrician	Drywall Finisher and Installer	Solar Panel Installer
Elevator Installer and Repairer	Equipment Operator (Heavy Machinery)	Stonemason
Exterminator	Excavator Operator	Structural Iron and Steel Worker
Fitter	Exterminator (Pest Control Technician)	Tire Technician
Gas Appliance Repairer	Flooring Installer	Tool and Die Maker
Gearbox Specialist	Furniture Finisher	Tower Climber & Maintenance Technician
Generator Mechanic	Glazier	Upholsterer
Janitor	Handyman	Vehicle Maintenance Supervisor
Locksmith	Hazardous Materials Removal Worker	Wastewater Treatment Operator
Maintenance & Repair Worker	Heavy Equipment Mechanic	Welder
Construction Electrician	Heating, Ventilation, and AC Technician	Window Installer
Motorcycle Mechanic	Industrial Machinery Mechanic	Woodworker
Painter	Caterer	Wind Turbine Technician
Plumber	Fashion Designer	Dress Maker

### Requirements: Technical, Vocational Construction, Maintenance, and Repairs

Most workers in this field of careers have a minimum of JHS or SHS. Others learn or attend either a technical or a vocational school, whiles others have degrees from university. Mostly the fitters learn their skills through on-the-job training.

### Engineering Science - Engineering, Innovation and Mathematics Studies

The Engineering Science - Engineering, Innovation, and Mathematics Studies, provide the basic educational knowledge of a broad interdisciplinary field that integrates principles of engineering, scientific methods, and mathematical analysis to solve real-world complex technical problems by focusing on core concepts in physics, chemistry, materials science, and mathematics. Engineering Science focuses on applying scientific principles to real-world engineering problems. Bridge the gap between theoretical science and practical engineering applications such as Aerospace, Biological, Civil, Chemical, Computer, Environmental, Electrical and Electronics. Mathematics for Engineering provides the quantitative tools needed for problem-solving and system modeling.

### Engineering Science, Innovation and Mathematics Careers

Professional in Engineering Science and Mathematics field like mechanical, electrical, civil, and chemical engineering have the ability, knowledge, experience and problem-solving skills. They develop analytical and critical thinking skills to tackle engineering challenges. Using Innovation and Research to supports technological advancements in emerging areas like renewable energy, robotics, and Artificial Intelligence (AI). Careers in Engineering and Mathematics is shown in Table 8, and offer alternative careers options.

**Table 8.** Engineering Science - Engineering, Innovations and Mathematics Careers.

Acoustical Engineer	Construction Electrician	Meteorologist
Actuary	Control Systems Engineer	Mining Engineer
Aerospace Engineer	Control/Instrumentation Engineer	Molecular Biologist
Aerospace Technician	Cryptographer	Nanoengineer
Aircraft Mechanic	Cybersecurity Engineer	Network Engineer
Aircraft Systems Engineer	Data Analyst	Nuclear Engineer
Architect	Data Scientist	Oceanographer
Architectural Drafter	Design Engineer	Operations Research Analyst
	Draftsman	Optical Engineer
Architectural Engineer	Ecologist	Petroleum Engineer
Architectural Technician	Electrical Engineer	Pharmaceutical Scientist
Artificial Intelligence Engineer	Electrical Engineering Technologist	Photonics Engineer
Astrobiologist	Electrician	Physicist
Astronomer	Electromechanical Equipment Assembler	Planetary Scientist
Astrophysicist	Electronics Drafter	Plasma Physicist
Automation Engineer	Electronics Engineer	Process Engineer
Automobile Mechanic	Energy Systems Engineer	Quantum Computing Scientist
Automotive Engineer	Environmental Engineer	Quantum Physicist
Biochemical Engineer	Environmental Scientist	Remote Sensing Scientist
Biofuels Processing Technician	Epidemiologist	Renewable Energy Engineer
Biological Scientist	Forensic Scientist	Research Scientist
Biological Technician	Genetic Engineer	Robotics Engineer
Biomass Plant Technician	Geologist	Satellite Engineer
Biomechanics Engineer	Geophysicist	Seismologist
Biomedical Engineer	Industrial Engineer	Software Engineer
Biostatistician	Information Security Analyst	Solar Energy Engineer
Broadcast Technician	Instrumentation Engineer	Statistician
Cartographer	Machine Learning Engineer	Structural Engineer
Cartographer (Map Maker)	Manufacturing Engineer	Surveyor
Chemical Engineer	Marine Engineer	Systems Engineer
Chemical Technician	Materials Engineer	Telecommunications Engineer
Chemist	Mathematician	Theoretical Physicist
Civil Drafter	Mechanical Drafter	Transportation Engineer
Civil Engineer	Mechanical Engineer	Waste Management Engineer
Civil Engineering Technician	Mechatronics Engineer	Water Resources Engineer
Computer Engineer	Metallurgical Engineer	Wind Energy Engineer

### Requirements: Engineering Science, Innovations, and Mathematics

Most engineers require basic strong foundation in Mathematics, Physics, Chemistry, Biology and Computer Science to pursue a degree in Engineering Science and Mathematics. Professionals in this field require Bachelor’s Degree (BEng/BSc), Master’s Degree (MEng/MSc), or PhD (Doctorate).

### Health Science - Health Care, Medicine, and Allied Health Studies

The Health Science - Health Care, Medicine, and Allied Health Studies, provide basics educational knowledge of scientific, social, and behavioral aspects of health care and wellness, focusing on the prevention, diagnosis, and treatment of diseases and the promotion of overall health. This interdisciplinary field of study integrates knowledge from biology, chemistry, medicine, psychology, and public health to improve individual and community health outcomes. HSS cover basics of Biomedical Sciences, Public Health, Clinical Medicine, Nursing and Allied Health Professions, Nutrition and Dietetics, Pharmacy and Pharmacology, Mental Health and Psychology, Health Informatics and basics herbal medicine **.** Health Science Studies are fundamental for advancing healthcare practices, improving patient outcomes, and promoting public health on a global scale.

### Careers in Health Care, Medicine, and Allied Health Services

Workers in this category provide a wide range of services, which help people live healthier and happier lives. They prevent, diagnose and treat illnesses. And in some case complex surgical operation or transplant could be performed. Table 9 provide several career options in the Health Science and Health care services.

**Table 9.** Health Science - Health Care, Medicine, and Allied Health Services Careers.

Acute Care Nurse	Health Information Technician	Pharmacist
Allergist and Immunologist	Immunologist	Physical Therapist
Anatomist	Laboratory Technician	Physician
Anesthesiologist	Magnetic Resonance Imaging Technologist	Physician Assistant
Anesthesiologist Assistant	Medical and Clinical Laboratory Technician	Physiotherapist
Anesthetist	Medical and Clinical Laboratory Technologist	Plastic Surgeon
Audiologist	Medical and Health Services Manager	Podiatrist
Biomedical Engineer	Medical and Public Health Social Worker	Psychiatric Nurse
Cardiologist	Medical Appliance Technician	Radiologist
Cardiovascular Technologist	Medical Assistant	Radiotherapist
Chemist	Medical Microbiologist	Registered Nurse
Chiropractor	Medical Officer	Speech-Language Pathologist
Clinical Neuropsychologist	Medical Practitioner	Surgeon
Clinical Nurse Specialist	Medical Record	Traditional Birth Attendant
Clinical Psychologist	Medical Records Technician	Urologist
Clinical Research Coordinator	Medical Scientist	Veterinarian
Community Health Worker	Medical Secretary	Ward Assistant and Ward Help
Critical Care Nurse	Medical Transcriptionist	Mental Nurse
Dental Assistant	Mental Health Counselor	Midwife
Dental Hygienist	Mental Nurse	Neurodiagnostic Technologist
Dental Laboratory Technician	Midwifery	Neurologist
Dentist	Neurodiagnostic Technologist	Neuron physiologist
Dermatologist	Neurologist	Neuropathology
Diagnostic Medical Sonographer	Neuron Physiologist	Neuropsychologist
Dietetic Technician	Neuropathology	Neurosurgeon
Dietitian	Neuropsychologist	Nuclear Medicine Physician
Dietitian and Nutritionist		Nuclear Medicine Technologist
Echo Cardiographer	Nuclear Medicine Physician	Nurse
Emergency Medical Technician	Nuclear Medicine Technologist	Obstetrician
Endocrinologist		Occupational Therapist
Endoscopy Technician	Obstetrician	Ophthalmic Optician
Epidemiologist		Ophthalmologist
Etiologist	Ophthalmic Optician	Opticians
Family and General Practitioner		Optometrist
Gastro Entomologist		
General Dentist		
Geriatrician	Orthopedist	Pharmacist
Gynecologist	Pediatrician	Physical Therapist
		Physician

### Requirements: Health Care, Medicine, Allied Health Services

Preparation towards health care careers can take many years of studies. In most cases a Bachelor’s degree has to be obtained before the actual medical training begins. In other areas a Bachelor’s, Master’s or Associate degrees has to be obtained. And most healthcare assistance may have to attend specialized institutions to acquire a degree or diploma.

### Hospitality and Tourism - Hospitality, Hotelier, Travel and Tourism Studies

The Hospitality and Tourism - Hospitality, Hotelier, Travel and Tourism Studies, is to provide the basic educational knowledge of Hospitality and tourism industries, such as hotels, restaurants, travel agencies, event planning and tourism management. It provides the framework of the importance of customers and perspective of global tourism and travel trend and destination management.

### Careers in Hospitality, and Tourism, Hotelier, and Travel and Tour

People who work in the field of tourism and hospitality provide services that help people to enjoy their vacations and leisure times. Some hospitality services plan their activities, which include recreational services. Other makes the guest happy while they enjoy live programs and services. Table 10 is the list of careers in the Hospitality, and Tourism industries.

**Table 10.** Hospitality and Tourism - Hospitality, Hotelier, Travel and Tourism Careers.

Accommodation Manager	Exhibition Coordinator	Restaurant Host/Hostess
Adventure Tourism Guide	Flight Attendant	Restaurant Manager
Airline Customer Service Agent	Food and Beverage Director	Revenue Manager
Airline Reservation Agent	Food Critic	Ski Resort Manager
Airport Duty Manager	Food Safety Inspector	Sommelier (Wine Expert)
Airport Manager	Food Stylist	Spa Manager
Baker	Front Desk Agent	Sports Tourism Manager
Banquet Manager	Front Desk Clerk	Sustainable Tourism Specialist
Bartender	Guest Experience Manager	Theme Park Manager
Bed and Breakfast Owner	Hospitality Consultant	Tour Guide
Bellhop/Bell Attendant	Hospitality Trainer	Tour Operator
Butler	Hotel General Manager	Tourism Marketing Manager
Caterer	Recreational Manager	Tourism Research Analyst
Catering Manager	Hotel Manager	Travel Agent
Chef	Hotel Receptionist	Travel Blogger
Concierge	Hotel Reservation Agent	Travel Consultant
Conference and Event Coordinator	Hotelier	Travel Influencer
Cook	Housekeeper	Travel Photographer
Corporate Travel Manager	Housekeeping Manager	Travel Show Host
Cruise Director	Lodge Manager	Vacation Rental Manager
Cruise Line Manager	Luxury Travel Advisor	Waiter and Waitress
Cruise Ship Casino Dealer	Night Auditor (Hotel Accounting)	Waiter/Waitress
Cruise Ship Entertainer	Park Ranger (Eco-Tourism Sector)	Wellness Retreat Coordinator
Cruise Ship Staff	Personal Chef (Luxury Hospitality)	Wildlife Tour Operator
Culinary Instructor	Recreation Manager	Wine Tour Guide
Destination Manager	Resort Activities Coordinator	Yacht Crew Member
Eco Tourism Guide	Resort Manager	Yacht Steward/Stewardess
Event Planner	Restaurant Host and Hostess	

### Requirements: Hospitality, and Tourism, Hotelier, Travel and Tour Careers

At least high school education is required in most tourism and hospitality services. Travel and tour services also prefer people with vocational education, technical, college, polytechnic or university graduate.

### Law and Legal Systems - Law, Legal Systems, and Human Rights Studies

The Law and Legal Systems - Law, Legal Systems, and Human Rights Studies, is to provide basic educational knowledge involve in the exploration of legal principles, institutions, and practices that govern societies. This field encompasses analysis of laws, the role of legal institutions, constitutions and the impact of legal systems on individuals and communities. It is essential for understanding the frameworks that maintain order, protect rights, and resolve disputes. The Course provides foundation of law, civil law, criminal law, public law, international law, corporate and commercial law, dispute resolution, legal systems and institutions. The basics of Law and Legal practice are critical for training legal professionals, shaping public policy, and understanding the legal foundations that support justice and governance in society.

### Careers in Law, Legal Systems, and Human Rights

Workers in these categories help people to protect and preserve their rights and freedom. Law is one of the basic social institutions without which no society could exist if people could do just what they pleased. Within every society each person has some obligation towards one another. Table 11 provides the list of careers in Law, and Legal Practice.

**Table 11.** Law and Legal System - Law, Legal System and Human Rights Practice Careers.

Adjudicator	E-Discovery Specialist	Legal Operations Manager
Administrative Law Judge	Hearing Officer	Legal Secretary
Arbitrator	Human Rights Lawyer	Legal Technology Specialist
Attorney	Immigration Lawyer	Legislative Counsel
Attorney General	Judge	Legislator
Bailiff	Judicial Law Clerk	Magistrate
Civil Rights Advocate	Law Clerk	Magistrate Judge
Compliance Officer	Law Librarian	Mediator
Conciliator	Lawyer	Paralegal
Contract Manager	Legal Assistant	Professional Support Lawyer
Corporate Counsel	Legal Consultant	Prosecutor
Court Clerk	Legal Contractor	Title Examiner, Abstractor, and Searcher
Court Interpreter	Legal Operations Analyst	Victim Advocate
Court Reporter		

### Requirements: Law, Legal Practice and Human Right Support Services

Most lawyers have college education and degree from a Law schools and universities. Most judges have been lawyers. Workers in the paralegals usually have an associate or a bachelor’s degree, and may have specialized legal training.

### Life, and Physical Science Studies

The Life, and Physical Science Studies (LPSS), provide the basic educational knowledge of a wide range of disciplines that explore the natural world, from the molecular mechanisms of life, to the fundamental principles governing the universe. These fields are crucial for advancing scientific knowledge, technological innovation, and understanding the complexities of the environment and living organisms. Life Science Studies include Biology, Biochemistry, Botany, Zoology, Microbiology. While Physical Science Studies covers Physics, Chemistry, Earth Science and Astronomy. LPSS are foundational for scientific discovery and innovation, addressing global challenges, and improving the quality of life through a deeper understanding of natural phenomena.

### Life, and Physical Science Careers

Careers within these categories, mostly scientists, their discoveries and redevelopment help us to understand the functions of the world in which we live. There are areas where prevention is needed and where new development is required. Life, Physical, and Social Science careers in Table 12, offers option of career types in this category.

**Table 12.** Life and Physical - Life, and Physical Science Careers.

Agricultural Scientist	Environmental Economist	Forensic Scientist
Agronomist	Environmental Engineer	Hydrologist
Animal Scientist	Environmental Scientist	Marine Biologist
Anthropologist	Epidemiologist	Materials Scientist
Archaeologist	Geneticist	Meteorologist
Astronomer	Geochemist	Microbiologist
Astrophysicist	Geochronologist	Neuroscientist
Atmospheric Scientist	Geodetic Surveyor	Oceanographer
Bacteriologist	Geographer	Paleontologist
Biochemist	Geologist	Physicist
Biologist	Geomorphologist	Political Scientist
Biophysicist	Geophysicist	Psychologist
Biotechnologist	Geoscientist	Sociologist
Botanist	Biological Technician	Soil Scientist
Climatologist	Cartographer	Statistician
Conservation Scientist	Chemical Technician	Survey Researcher
Cosmologist	Chemist	Urban and Regional Planner
Economist	Ecologist	Zoologist
Environmental Compliance Inspector		

### Requirements for Life, Physical Sciences Careers

Most scientists have doctorate degree. A minimum of bachelor’s degree is required. In most cases these scientists and doctor of philosophy (PhD) undertake research with research assistance.

### Management - Management, Leadership, Politics, and Authority Studies

The Management, Leadership, Politics, and Authority Studies (MLPAS), provide the basic educational knowledge which delve into the principles and practices of guiding organizations, influencing political systems, and exercising power and authority. These fields are crucial for understanding how societies are organized, decisions are made, and leadership is exercised across various sectors. Management studies cover business, organizational behavior, financial management. Leadership ensures ethics, local and global politics, and international relations. Authority studies include sociology of power, legal authority and public administration. MLPAS are vital for equipping individuals with the knowledge and skills to lead organizations, influence public policy, and understand the complex dynamics of power in society.

### Management, Leadership, Politics, and Authority Careers

MLPAS workers, called managers are the leaders of businesses and organizations. All businesses and organizations need managers to plan and administer activities and policies and to train and supervise other employees. Some managers are elected officials, they develop policies and laws and direct activities. The levels of management are different with different amount of authority and responsibility. Upper managers and chief executive officers, president and vice president have the most authority. Table 13 provide an option of careers in the Management, Leadership, Politics, and Authority.

**Table 13.** Management - Management, Leadership, Politics, and Authority Careers.

Accountant	Director of Operations	Plant Foreman
Ambassador	Economic Development Director	Police Chief
Campaign Manager	Education Administrator	Policy Advisor
Center Manager	Executive Director	Political Analyst
Chief Executive Officer (CEO)	Foreman/Forewoman	Politician
Chief Financial Officer (CFO)	General Manager	President (of organizations or countries)
Chief Information Officer (CIO)	Governor	Press Secretary
Chief Marketing Officer (CMO)	Human Resources Manager	Product Manager
Chief Operating Officer (COO)	Immigration Officer	Project Manager
Chief Technology Officer (CTO)	Judge	Public Health Administrator
City Manager	Legislative Assistant	Public Relations Manager
City Planner	Manager	Regulatory Officer
Community Service Manager	Mayor	School Superintendent
Consultant	Military Officer	Social and Community Service Manager
County Administrator	Nonprofit Manager	State Representative
Customs and Border Protection Officer	Operations Manager	Supervisory Manager
Diplomat	Parliamentarian	

### Requirements: Management, Leadership, Politics, and Authority

Most managers, elected officials and management analysts are college graduates and many have advance degrees. Courses in business administration are helpful. Some organizations offer formal training programmes for their managers.

### Home Science - Home Economic, and Personal Services Studies

The Home Science - Personal Services, and Home Economics Studies (PSHES), provide the basic educational knowledge that focuses on skills and knowledge related to individual well-being, family management, and consumer services. These fields prepare individuals for careers in service-oriented industries and equip them with practical skills for managing personal and household needs. Personal Services include Cosmetology and Beauty, Fitness and Personal Training, Health and Wellness. Home Economics covers Family and Consumer Sciences, Textiles and Clothing, and Home Management, Food and Nutrition. Personal Services and Home Economics Studies are integral for preparing individuals to provide essential services that enhance quality of life, foster healthy living, and support personal and family well-being.

### Careers in Personal Services, and Home Economic

Some of the workers in this category require special skills to perform personal task for people. Many personal services are the type of services people can do themselves but for one reason or the other they prefer that specialist in these areas perform these tasks. Careers in Personal Services, and Home Economics in listed in Table 14.

**Table 14.** Home Science - Home Economics and Personal Services Careers.

Animal Caretaker	Hairstylist	Family and Consumer Sciences Educator
Animal Trainer	Home Care Aide	Home Economist
Barber	Home Health Aide	Massage Therapist
Beautician	Janitor and Cleaner	Personal Shopper
Bridal Consultant	Makeup Artist	Tailor/Dressmaker
Clergy	Manicurist	Laundry and Dry-Cleaning Worker
Concierge	Marriage and Family Therapist	Social and Community Service Manager
Cosmetologist	Nanny	Event Planner
Embalmer	Pedicurist	Butler
Fitness Trainer	Child Care Provider	Sommelier
Flight Attendant	Dietitian/Nutritionist	Tour Guide
Floral Designer	Interior Designer	Personal Assistant
Funeral Director	Culinary Professional	Life Coach
Hairdresser		

### Requirements: Personal Services, and Home Economics

Most workers in this field require formal education or training. However, at least Junior school level may be required, other workers receive on the job training, some may have to attend special training and have degree.

### Industrial Technology - Production, Manufacturing and Industrial Studies

The Industrial Technology - Production, Manufacturing, and Industrial Studies (PMIS), provide the basic educational knowledge of broad field that encompasses various aspects of creating goods and managing industrial processes. Production teaches the process of transforming raw materials into finished goods through various methods and processes. Manufacturing provides steps through which raw materials are transformed into a final product. Industrial Studies is an interdisciplinary field that examines industrial processes, systems, and organizations to improve efficiency, productivity, and sustainability. Technological Integration provide the use of control systems for operating equipment. Applications of Health and Safety in addition to Research and Development (R&D). This field is dynamic and continuously evolving with advancements in technology and shifts in global markets, making it a vital area of study for improving industrial efficiency and innovation.

### Careers in Production, Manufacturing, and Industry

Workers in this category are involved in making or preparing goods either by hand or machine. These products range from simple wooden objects such as tables and chairs to complex computer parts. Some production workers are involved in food processing. Production, Manufacturing, and Industrial careers options is provided in Table 15.

**Table 15.** Industrial Technology - Industrial, Manufacturing, and Production Careers.

AI and Data Analyst	Logistics Coordinator	Chemical Plant Operator
Assembler	Machine Operator	Food Processing Operator
Assembly Line Worker	Machinist	Metal and Plastic Machine Worker
Baker	Manufacturing Engineer	Ophthalmic Laboratory Technician
Bindery Worker	Manufacturing Technician	Painting and Coating Worker
Butcher	Meat Dresser	Power Plant Operator
CNC Machinist	Millwright	Semiconductor Processor
Desktop Publisher	Mine Worker	Sewer and Tailor
Detailer	Precision Assembler	Slaughterer and Meat Packer
Dressmaker	Prepress Worker	Textile Worker
Industrial Electrician	Printing Press Operator	Tool and Die Maker
Environmental Engineer	Procurement Specialist	Welder
Industrial Automation Specialist	Production Manager	Woodworker
Industrial Designer	Quality Control Specialist	Quality Engineer
Industrial Hygienist	Robotics Technician	Process Engineer
Inventory Manager	Safety Officer	Lithographer
Jeweler	Boilermaker	

### Requirements: Production, Manufacturing, and Industry

Most workers in this group have to study at college, technical or vocational school. Others pursue apprenticeship programs. In other area, JHS or SHS level is required, while in other sectors formal education and Bachelor’s or Master’s degree is required.

### Business and Finance - Business, Accounting, Banking, and Financial Studies

The Sales, Business, Accounting, Banking, and Financial Studies, Provide the basic educational knowledge of a comprehensive field that covers various aspects of commerce, finance, and economic transactions. Sales and process of promoting and selling goods or services. Business activity of making, buying, or selling goods or services for profit. Banking the business conducted or services offered by a bank, including holding money for savings and checking accounts or issuing loans. Financial studies of how individuals, businesses, and organizations manage and use financial resources. This field is vital for understanding the dynamics of how businesses operate, how sales drive growth, and how financial systems underpin the economy, providing a foundation for careers in various industries.

### Careers in Business, Accounting, Banking, and Financial Services

Workers in the areas of sales, service, Banking and finance inform people about products or services and persuade them to buy. Most sales workers sell their products in the markets and in shops and super markets. There are other sales representatives ready to answer questions about a product or to demonstrate how a product works. Careers in Sales, Business, Banking, and Finance options in listed in Table 16.

**Table 16.** Business and Finance - Business, Accounting, Banking and Financial Careers.

Account Executive	Financial Planner	Telemarketer
Actuary	Insurance Sales Agent	Treasury Analyst
Advertising Sales Agent	Investment Banker	Wholesaler
Banker	Manufacturer’s Representative	Budget Analyst
Branch Manager	Marketing Manager	Accountant
Business Analyst	Operations Manager	Loan Officer
Business Development Manager	Real Estate Agent	Credit Analyst
Entrepreneur	Retail Banker	Financial Manager
Commercial Banker	Retail Sales Associate	Tax Preparer
Consultant	Risk Manager	Claims Adjuster
Data Scientist	Sales Manager	Insurance Underwriter
E-Commerce Specialist	Sales Representative	Financial Quantitative Analyst
Financial Analyst	Salesforce Administrator	Financial Risk Specialist
Businessman/Businesswoman	Stockbroker	

### Requirements: Business, Accounting, Banking, and Finance Careers

Companies that employ sales workers prefer to have at least high school education. Usually, they receive on-the-job training. Insurance sales agents, stockbrokers and other financial services employ people with a degree or college education. Wholesale, retail and manufacturing sale representatives prefer to have people with college or university degree.

### Sports and Music - Sports, Music, Acting and Entertainment Studies

The Sports, Music and Entertainment Studies (SMES), provide the basic educational knowledge of interdisciplinary field which focuses on preparing students for careers in sports, music and entertainment industries. The course provides combine creative technical and artistic skills. Sports management, athlete representation, music production, concert and event promotion, Film, Radio and Television production.

### Careers in Sports, Music, Acting, and Entertainment

Millions of people are actively involved in sporting events and enjoy the excitement of competition. Entertainment activities amuse many audiences. Others, like beauty contests and concert performances, are also enjoyed by thousands of people. Some workers specialized in certain sporting and athletic activities. Sports, Music, and Entertainment career options are provided in Table 17.

**Table 17.** Sports, Music - Sports, Music, Acting, and Entertainment Careers.

Acoustician	Cyclist	Goalkeeper
Actor/Actress	Dancer	Music Director
Amusement Attendant	Director (Television, and Radio)	Screenwriter
Athlete	Disc Jockey	Set Designer
Balloonist	Entertainer	Sound Engineer
Bandleader/Bandmaster	Fashion Coordinator	Sports Announcer
Bandsman	Fashion Designer	Sports Official/Referee
Baseball Player	Fashion Editor	Stunt Performer
Basketball Player	Fashion Photographer	Talent Agent
Choreographer	Fashion Writer/Artist	Theater Manager
Coach	Film Director	Video Game Designer
Comedian	Film Laboratory Technician	Voice-over Artist
Composer	Fitness Trainer	Wardrobe Stylist
Conductor	Footballer	Wedding Planner
		Yoga Instructor

### Requirements: Sports, Music, and Entertainment Careers

For many careers in the sports and entertainment group, there are no specific educational requirements before one cold engage in such activities. Most of the people engaged in sports or entertainment have natural talent and they practice or train for hours in a day. Many athletes earn degrees while they compete in college athletics programs. Officials of sports were in most cases sports personnel and may specialize in coaching or as trainers. Other attends colleges or earns degree from university.

### Education - Education, Training Teaching and Learning Studies

The Education – Education, Training, Teaching, and Learning studies to offer basic educational knowledge of teaching, training and education studies as academic and professional field focused on the theory and practice of facilitating learning and skill development, as pre-requisite entry requirement to teacher training colleges.

### Careers in Teaching, Education, and Training

Workers in this professional group offer systematic information to students based on subjects. They teach many kinds of skills and cultural values to students of all ages. A variety of methods of teaching are employed, including practical, laboratory, workshops, presentations, demonstrations, excursions, research, the use of computers and information technology, and project presentations. While others practice a Dual Education System. Career options in Teaching, Education, and Training are provided in Table 18.

**Table 18.** Education - Education, Training, Teaching and Learning Careers.

Adapted Physical Education Specialist	Education Teacher (Postsecondary)	Library Science Teacher (Postsecondary)
Adult Literacy and Remedial Education Teacher	Elementary School Teacher	Life, and Physical Science Studies Teacher
Administration, and Office Support Teacher	Engineering Science, and Mathematics Studies Teacher	Management, Leadership, Politics, and Authority Studies Teacher
Agricultural Science Teacher (Postsecondary)	Engineering Teacher (Postsecondary)	Mathematical Science Teacher (Postsecondary)
Anthropology and Archaeology Teacher (Postsecondary)	English Language and Literature Teacher	Montessori Teacher
Architectural Teacher (Postsecondary)	Environmental Science Teacher (Postsecondary)	Music Instructor (Private & Institutional)
Area, Ethnic, and Cultural Studies Teacher	Forestry and Conservation Science Teacher	Personal Services, and Home Economics Studies Teacher
Art, Drama, and Music Teacher	Geography Teacher	Physical Education Teacher
Art, Media, Communication, and Design Teacher	Health Science Studies Teacher	Physics Teacher (Postsecondary)
	Head Teacher	Preschool Teacher
Atmospheric, Earth, Marine, and Space Science Teacher	Headmaster/Headmistress	Principal/School Administrator
Biological Science Teacher (Postsecondary)	Health Specialties Teacher (Postsecondary)	Production, Manufacturing, and Industrial Studies Teacher
Business Teacher (Postsecondary)	History Teacher (Postsecondary)	Psychology Teacher (Postsecondary)
Chemistry Teacher (Postsecondary)	Home Economics Teacher (Postsecondary)	Sales, Commerce, Banking, and Financial Studies Teacher
College and University Faculty	Hospitality, and Tourism Studies Teacher	Science Teacher (General)
Communications Teacher (Postsecondary)	Instructional Coordinator	Secondary School Teacher (SHS)
Community, Civil Protection, and Social Studies Teacher	Junior High School Teacher (JHS)	Special Education Teacher
Computer Science Teacher (Postsecondary)	Kindergarten Teacher	Sports, Music, and Entertainment Studies Teacher
Construction, Maintenance, Repairs, and Technical Studies Teacher	Law, and Legal Systems Studies Teacher	Teacher Trainer/Educational Consultant
Continuing Education Teacher	Law Teacher (Postsecondary)	Teaching Assistant
Criminal Justice/Law Enforcement Teacher	Transportation, and Facilities Studies Teacher	Teaching, Education, and Training Studies Teacher
Early Childhood Education Teacher	Tutor/Private Educator	Technical and Vocational Education Teacher
Economics Teacher (Postsecondary)		Technology, IoT, Computer, and Coding Studies Teacher

### Requirements: Teaching, Education, and Training

Almost all teachers need a bachelor’s degree. Others are required to complete a professional teacher training program before they can teach. Some pre-school teachers and teacher’s aides do not need a degree. Many high school teachers and most college and university faculty have advanced degrees. Those teaching in the universities have a doctorate degree others require a minimum of a master’s degree.

### Computer Science – Computer, Technology, IoT, and Coding Studies

The Computer Science – Computer, Technology, IoT, and coding Studies focuse on the basic educational knowledge of understanding, designing and applying mordern technological systems and programming languages of coding to solve real word problems. Study tools and systems such as Artificial Intelligence (AI), Cybersecurity, Data Science, Cloud Computing and Human-Computer Interraction (HCI).

### Careers in Computers, Technology, Internet-of-things, Computer, and Coding

Workers in this group use computer and technology to prepare or analyze a variety of complicated procedures. Business and organization cannot function efficiently without these professionals who design complicated items like the microprocessor clip, computers, automobile, aircraft and other complex projects. Careers in the Technology, Internet-of-things, Computer, and Coding in provided in Table 19.

**Table 19.** Computer Science - Computer, Technology, IoT, and Coding Studies Careers.

Actuaries	Computer Systems Analyst	Information Security Analyst
AI Research Scientist	Computer Technician	IoT Architect
Artificial Intelligence Specialist	Computer/Office machine Technician	IoT Developer
Augmented Reality (AR) Developer	Cybersecurity Analyst	Machine Learning Engineer
Blockchain Developer	Data Analyst	Mobile Application Developer
Cloud Engineer	Data Scientist	Network Engineer
Cloud Solutions Architect	Database Administrator	Robotics Engineer
Computer Network Architect	Database Architect	Systems Analyst
Computer Programmer	DevOps Engineer	User Experience (UX) Designer
Computer Security Specialist	Embedded Systems Engineer	Virtual Reality (VR) Developer
Computer Software Engineer	Game Developer	Web Developer
Computer Support Specialist	Hardware Engineer	

### Requirements: Technology, Internet-of-things, Computer, and Coding

Almost all the professionals in the fields of Computer Science and Technology, either have to complete their education at a technical institution, college, polytechnic or university. Most employers require a bachelor’s degree.

### Transportation - Aviation, Shipping, Railways and Vehicular Studies

The Transportation - Aviation, Shipping, Railways and Vehicular Studies, provide basic educational knowledge in planning, design, management and optimization of transportation systems and facilities. It emphasizes sustainability, efficiency and safety. The transportation sector include; Air, Rail, Road, and Water transportation. The study provides knowledge acquisition in the systems and network of Aviation, Aircraft and Airport systems. It also includes; Maritime, Shipping, Harbour, Port freight and forward, import and export systems, railway and train service system, Heavy duty vehicles and light trucks, commercial Buses and private cars used to move people, goods and services efficiently and safely.

### Careers in Transportation - Aviation, Shipping, Railways and Vehicular Studies

People, goods and services moving from one place to another depend greatly on transport services. These services range from air, sea, rail and road transportation, worker in the field of transport help passengers and goods to move from one place to another. Transportation, and Facilities careers Table 20 provide an option of water, rail, road and air transportation.

**Table 20.** Transportation - Aviation, Shipping, Railways and Vehicular Careers.

Able Seaman	Aircraft Systems Assembler, Precision	Commercial Pilot
Agricultural Machinery Operator	Aircraftman	Construction Equipment Operator
Air Chief Marshal	Airfield Operations Specialist	Automotive Mechanic
Air Crew Member	Airframe-and-Power-Plant Mechanic	Logistics Analyst
Air Crew Officer	Airline Pilot, Copilot, and Flight Engineer	Logistics Specialist
Flight Attendant	Airman or Airwoman	Transportation Analyst
Air Marshal	Ambulance Driver	Import/Export Clerk
Air Traffic Controller	Boatswain (Bosun)	Dispatcher
Air Vice-Marshal	Bulldozer Operator	Warehouse Associate
Aircraft Body & Bonded Structure Repairer	School Bus Driver	Quality Control Clerk
Aircraft Cargo Handling Supervisor	Transit and Intercity Bus Driver	Traffic Office Specialist
Aircraft Engine Specialist	Bus, Truck and Diesel Engine Specialist	Railroad Worker
Aircraft Launch and Recovery Officer	Captain	Ship Engineer
Aircraft Launch and Recovery Specialist	Car and Wagon Driver	Logistician
Aircraft Mechanic and Service Technician	Chief Engineer	Captain (Maritime)
Aircraft Rigging Assembler	Chief Officer	Logistics Manager
Aircraft Structure Assembler, Precision	Commercial Diver	Aircraft Structure, Rigging, and Systems Assembler

### Requirements: Transportation - Aviation, Shipping, Railways and Vehicular Studies

At least all qualified drivers should have basic formal education and should possess valid driver’s license. Aircraft pilot should have bachelor’s degree. Many water transportation workers have professional training or bachelor’s degree from Maritime University or nautical college. Air transportation requires special training relating to aviation and airport systems.

## Step 6: Evaluation and Feedback

Evaluation and Feedback of CCSP-18 to promoting EFA as educational and curriculum reforms is needed to assess progress and performance, or output of CCSP-18 and provide responses for improvement or recognition of CCSP-18 for EFA. Where concepts such as CCSP-18 could have systematic process to measure the quality, effectiveness, or performance of Examination and assessment body, Teaching and supervisory system, Student and pupils’ evaluations, teaching materials and learning outcomes of CCSP-18 for EFA.

Feedback from the stakeholders; Examination and assessment bodies, Teaching and supervisory system, Student and pupils on the educational and curriculum reforms for CCSP-18 for EFA. Feedback communicates the evaluation results and suggests actions to improve or sustain performance of CCSP-18 for EFA.

## Conclusions

The challenges in choosing a course of study in a Senior High School in Ghana as a foundation to a student’s career aspiration has always been a challenge for students, parents, guardians and the school authorities due to the policy of limited number of the main Courses Offered by the Ministry of Education, the Ghana Education Service and the West Africa Examinations Council. This problem calls for a reform in Ghanaian education, because knowledge and technology have advanced and teaching and learning has evolved. The introduction of new Education Reform using Common Career Selective Programme (CCSP) to promulgate Education-for-All (EFA) provide the key to solving these problems.

The CCSP provided 18 selective Core subjects known as CCSP-18, with course header, course descriptions, specified courses option, elective courses, career options, career requirements and a list of tables of related careers. The CCSP-18 concept offer students a diverse range of course options to drive individual natural capabilities and future career aspirations for lifelong learning.

The significance of the study is that the basic educational knowledge acquired during Senior High School and Senior High Technical Schools provide seamless transition of knowledge from the basic to advance level of education to the world of work. The policy brief outlines policy outcomes and implications, and actionable recommendations in terms of Courses Offered, Curriculum requirements and career guidance.

In conclusion, the CCSP-18 for EFA concept has the potential to affect the lives of millions of students globally in choosing a course of study, as the key to unlock students’ natural potentials in terms of their abilities, interest, aspiration and dreams, in choosing a course of study, to drive development of Education-for-All.

## Recommendations

• Domestic and Regional stakeholders and collaborators to reform the current educational system, develop the necessary curriculums for adoption and implementation CCSP-18 for EFA.
• Model schools equipped with the necessary facilities and resources to experimentally implementation CCSP-18 for EFA as an action to drive development framework of EFA by 2030.
• Facilitator for CCSP-18 for EFA to begin modernization of teaching and learning to enhance their knowledge and skills using Competent Base Training (CBT).
• Method of teaching for CCSP-18 for EFA could be based on 80% for all Academic work and 20% hands-on or internship programmes, and 80% practical’s and 20% theory for all trade and apprentice programmes.

## Data Availability

The data used for the study was based on electronic copy of Ministry of Education, Ghana Education Service, Ghana Senior High Schools Annual Digest 2019/2020 ( https://ges.gov.gh) Annual-Digest-2020-ecopy.pdf. The number of Courses Offered and School Statistics were tabulated from the “Annual Digest 2019/2020”. The data tabulated from “2021 Second Cycle Schools Register” for the Academic Year 2019/2020, where the numbers of Courses Offered and the Students Statistics of the number of students in (SHS 1), (SHS 2), and (SHS 3).
