# Education Reform using Common Career Selective Programme (CCSP) to Promote Education-for-All (EFA)

**DOI:** 10.12688/f1000research.163862.2

**Published:** 2025-10-29

**Authors:** Frederick Agyemang, Benedicta Woode, Samuel Kofi Fosuhene, Nana Ama Browne Klutse, Alexander Kojo Nyarko, Seth Oku Asamoah, Bismark Antwi Agyei

**Affiliations:** 1 Ghana Atomic Energy Commission (GAEC), Accra, Greater Accra Region, Ghana; 2 Wisdom and Instruction Career Advisory Board (WICAB), Accra, Ghana; 3 University of Ghana (UG), Accra, Greater Accra Region, Ghana; 4 Ghana Education Service (GES), Accra, Ghana; 5 Academy for Advanced and Further Education and Training (AFAFET) Global, Accra, Ghana

**Keywords:** Education Reform, Education-for-All (EFA), Pre-Tertiary Education, Career Guidance, Common Career Selective Programme (CCSP), career and technical, Curriculum, high school, elementary school, middle school

## Abstract

Choosing a course of study in a Senior High School in Ghana is a major problem for both students and parents, due to the limited number of Courses Offered. The courses are governed by the national education and curriculum framework. The educational policy guideline provided the main courses as: Agriculture, Business, Technical, Home Economics, Visual Arts, General Arts, and General Science options, irrespective of individual career aspiration. A report made public in 2018 indicated that, education in Ghana is not of good quality, students attend school from basic to secondary level for an average of 12 years, only at half capacity. The outcome of the poor quality of the education system is waste of human capital resources. To address these challenges, the study explored education reform and new policies that brings systematic changes across the entire education system, particularly in the design of courses and curricula. The study employed content analysis based on the Ghana Education Service's 2021 second-cycle school register, and data analyzed from the Ghana Senior High Schools Annual Digest 2019/2020. The methodology employed Common Career Selective Programme, a structured basic education and training programme, designed to help improve the quality of education, and for students to explore within a broader curriculum. The proposal modified the seven main courses into 18 major courses, which consist of a course header, abstract, course description, career options and requirements. The results provided a variety of course options that suit individual innate abilities, focused on the student’s career goals, intensify lesson content, improve quality of education, and advance human resource capacity, providing Education-for-All beyond the year 2030. In conclusion, the study proposed education reform for courses and curricula, to promote basic education knowledge, actionable recommendations, and policy amendments to improve and promote quality education in Ghana, West Africa, and other countries globally.

## Introduction

The Government of Ghana (GoG) has initiated some key education reform under the Education Strategic Plan (ESP 2018-2030) ( Amegah & Iddrisu, 2024). The ESP is expected to contribute to the United Nations (UN) Sustainable Development Goals (SDG 4), Quality Education ( Boeren, 2019) which seeks “to ensure inclusive equitable quality education and promote lifelong learning opportunities for all”. The establishment of United Nations Educational, Scientific, and Cultural Organization (UNESCO), is aimed at building peace through education, science and culture, while International Bureau of Education (UNESCO-IBE) is to develop educational curriculums ( Jones, 2018). To this end the Incheon (South Korea) Declaration and the education 2030 framework for action is also committed to educating the community as the main driver of development ( Adu-Gyamfi et al., 2016). Teaching and learning have evolved, therefore the need for comprehensive education reform to make learning enjoyable, passionate, and aligned with career aspirations, in order to achieve lifelong individual career goals. In response to this, this policy brief proposes education reform to promote lifelong learning that ensures education is accessible to all.

### Research objective

The main objectives of this research is to analyze the gaps in the current education system that limit equal access and the relevance to career aspirations. Examine the potential of the Common Career Selective Programme (CCSP), as a framework for aligning curriculum with lifelong learning and employability. Assess how CCSP can support the goals of Education-for-All (EFA) by making education more inclusive, equitable, and career-oriented. To identify policy measures required to integrate CCSP into Ghana’s pre-tertiary education system. And to provide actionable recommendations for policymakers on implementing of CCSP to drive systematic education reform.

Specific objectives:

1. To evaluate the role of CCSP in addressing systemic gaps in Ghana’s education system.
2. To determine how CCSP can enhance inclusivity, equity, and career relevance in line with Education-for-All (EFA).
3. To propose policy recommendations for implementing CCSP as a strategy for sustainable education reform.

## Literature review

### Education reform

Education reform calls for new policies that bring about systematic changes across the entire structure of the education system, particularly in the design of courses and curricula. In Ghana, the Ministry of Education (MoE) together with the Ghana Education Service (GES) oversee policy formulation and the implementation of approved courses and programmes at the pre-tertiary level. The programmes are aimed at equitable quality formal education ( Adu-Agyem & Osei-Poku, 2012). Educational reforms also go with Standard Based Curriculum (SBC) ( Asante et al., 2024) development to attain education system for domestic and international recognition. Ghana's new educational reform over the years includes the introduction of Technical and Vocational Education and Training (TVET), which was hope to close the gap between theory and hands-on training, and Science, Technology, Engineering and Mathematics (STEM) to equip learners with the mindset and tools to solve problems, and innovate. The TVET review to assess its effectiveness in Ethiopia proposed practical implementation based on international best practice, and policy enactment across Africa ( Hagos Baraki & van Kemenade, 2013). Currently, TVET is been implemented in many Africa countries and beyond. Education reform requires collaboration of all stakeholders. Regional organization such as the West Africa Examination Council (WAEC) ( Nweze & Obu, 2022), an examination board mandated to harmonize standardized examination, and committed to excellent education, providing quality and reliable education assessment in the West Africa should be part of any major education reform.

Education in Ghana over the years has experienced education reforms ( Kuyini, 2013). The proposed new education reform requires holistic approach devoid of intermittent interference during regime change. The reforms should cover all the pre-tertiary schools: Preparatory schools’ education could mimic Montessori education, which involves the children’s natural intrinsic interest. Primary Schools to provide formative learning of literacy and numeracy. Junior High Schools (JHS) to provide foundation to specialized advance subjects. Senior High Schools (SHS), TVET and STEM schools to provide career development programmes, as an entry point to the world of work. While tertiary education and higher educational institutes (HEI) could concentrate on Research, Development and Demonstration (RD&D) ( Sweet & Palazzi, 2015) programmes tailored to fulfil lifelong Education-for-All (EFA).

Lifelong learning begins with theoretical understanding of personality inventory (PI), based on the Big-five factors of self-rating; Surgency, Agreeableness, Conscientiousness, Emotional Stability, and intellect ( Goldberg, 1993). Where bipolar and unipolar are useful in educational setting, to influence the correlation between course of study and career aspiration. The Holland RIASEC typology ( Holland, 1997), relate personal characteristics to occupational structures, where Self-Directed Search (SDS), and personal career theory (PCT) view three components of the PCT as: (1) personal characteristics, (2) occupational knowledge, or (3) translation units, which serves as guidance for education and career. The PCT is fundamentally a matching system, probably developed informally over a lifetime. However, when this rudimentary matching system stalls or fails, then Holland's career choice theory is based on matching one's career interests with suitable jobs. Where the RIASEC’s interest define combination of other additional interest measures, to identify how students from various educational settings and Vocational choice theory allows individuals to freely choose careers based on their interests. According to Holland, individuals are attracted to certain careers because of their personality, and a number of variables that constitute their background.

### Poor education in Ghana

The World Bank report in 2018 has warned that about 56 per cent of Ghana’s human capital will go waste in the 18 years because of the poor quality of the country’s education system. The Human Capital Index (HCI) measures the amount of capital that a child born in the country today can expect to attain by age 18. It stated that the poor quality of education would translate into lack of capacity to support sustainable national development. The report made public position Ghana 116 out of 157 countries. “The reality is that the education in Ghana is not of good quality”. Dr Guiffrida observed that although Ghana had enormous natural resources potential, the country had not been able to sustainably develop because of the weak capacity of human resources.

Ghana is the last but one country on the latest World Bank Human Capital Index (HCI), which looked at the performance of basic and secondary school candidate in harmonized test scores globally. The Poverty ravaged Niger was the only country Ghana was able to beat on the ranking with a score of 307 which was way below the average score of 391 for Lower Middle-Income (LMI) Countries. The five lowest-ranked countries which are all in Africa are Niger, 305; Ghana, 307; Mali, 307, Seirra Leone, 316; and the Democratic Republic of Congo, 318. Ghana’s low ranking could be blamed on the poor quality of education of education in the country at the basic and secondary schools’ level, the report stated.

“For Ghana, although children attend school for an average of 12 years from basic to secondary level, if you measure the results of their test scores, you will realize that the amount of content they have learnt is just equivalent to what someone in another country will learn in about 6 years. It means that young people are learning at only half capacity. Ghana’s enrolment is high, but what the children learn leaves so much to be desired” Henry Kerali, the Country Director of the World Bank office in Ghana explained. He added that the only way to reverse the trend is for the country to invest in its human resources to imbibe the right innovation and technology that would help make the best use of the natural resources.

### Policy outcomes and implications

The data analyzed from Ghana Senior High Schools Annual Digest 2019/2020, put the number of year one students (SHS 1) is 400,612. The number of year two students (SHS 2) is 426,219. And the number of year three students (SHS 3) at 354,126. The total number of students per one academic year is 1,180,957. The limited number of courses studied dwindles the human capacity index of the graduate students the schools produce. Annually, more than 350,000 SHS students graduate, only to be confronted with another challenge of choosing a course for further studies at a tertiary institution, different from the course they studied in the high schools. Again, most graduates pursue a career different from the courses they studied in the high schools, and their intrinsic individual abilities, career interests, and career goals are in variance. This problem requires a call-to-action for education reform and new curriculum.

Curriculum reforms globally adhered to the six-step approach to curriculum development ( Khan & Abu Salek, 2019). Step 1: Problem Identification and General Needs Assessment. Step 2: Targeted Needs Assessment. Step 3: Goals and Objectives. Step 4: Educational Strategies. Step 5: Implementation. And Step 6: Evaluation and Feedback.

## Methodology

### Common Career Selective Programme

To address the challenges of young people learning at half capacity of education in Ghana, the poor quality of education, and the problem of courses offered selection, the method adopted is course and curricula modification (CCM), to assess the potential of Common Career Selection Programme (CCSP), as an alternative solution to these challenges. The CCSP provides equal access of education using CCM, where the content of courses and curricula plays a key role in the education reform, to modernize and improve the quality of teaching and learning.

The CCSP concept is a modification of the 7 main courses into 18 Major courses (CCSP-18), as Courses Offered. It consists of Course Header, where every student is mandated to selected one out of the 18 courses. Courses Abstract, which provide a brief summary, as a quick overview of the major course. Courses Description, provide detail content of the major course, the main focus, goals, and scope of the course. Other requirements of the major courses include; Course Curriculums, Core Subjects, Selective Subjects, Elective Subjects, Language and Culture studies, Subject Combinations and others subject options for the major courses.

The CCM of the seven main courses are modified as follows:

1. **Agriculture Science** remain as **one** major course
2. **Business** is modified into **three** major courses
3. **Technical** is modified into **two** major courses
4. **Home Economics** is modified into **two** major courses
5. **Visual Arts** as **one** major course and renamed as Audio Visual and Physical Arts
6. **General Arts** is modified into **four** major courses
7. **General Science** is modified into **five** major courses

The CCSP is a structured basic educational knowledge and training programme, designed to help students to explore within a broader curriculum of education, for individuals to harness their innate abilities through the various career paths, under education and training within educational setting. Where the pedagogies of the Basic School, Senior High School, Senior High Technical School, Technical and Vocational Education and Training, and Science, Technology, Engineering and Mathematics schools, to deliver basic educational knowledge and training. In addition, the CCSP provides tabulated list of career options of the major courses, and career requirement for specific qualification, skills and experience as a condition to perform certain professions. The Table 1 shows modified courses and curricula for the CCSP.

**Table 1.** Courses and Curricula Modification for Common Career Selective Programme

Courses Offered	Common Career Selective Programme	Courses Abstract	Code
Agriculture	Agriculture Science	Farming, Fisheries, Forestry, Poultry, Animal Husbandry, and Food Production Studies	101
Business	Administration	Administration, Secretarial, and Office Support Studies	102
	Business and Finance	Business, Accounting, Banking, and Financial Studies	103
	Management	Management, Leadership, Politics, and Authority Studies	104
Technical	Technical and Vocational (TVET)	Technical, Construction, Repairs, and Maintenance, Fashion, Catering and Vocational Studies	105
	Production Technology	Industrial Processing, Machinery, Manufacturing, and Production Studies	106
Home Economics	Home Science	Home Economics, Personal Services, Fashion and Catering Studies	107
	Hospitality and Tourism	Hospitality, Hotelier, Travel and Tourism Studies	108
Visual Arts	Audio Visual, and Physical Arts	Audio Visual Arts, Performing Arts and Literary Arts, Sports, Music, and Design Studies	109
General Arts	Multimedia Arts	Radio and Television, Media, Communication, and Journalism Studies	110
	Law and Legal Arts	Law, Legal Systems, Human Rights and Constitutional Studies	111
	Education and Training	Education, Training, Teaching and Learning, and Faculty Studies	112
	Social Science	Humanities, Communities, Civil Protection and Social Studies	113
General Science	Health Science	Health Care, Medicine, and Allied Health Studies	114
	Engineering Science (STEM)	Engineering, Innovation and Mathematics Studies	115
	Life and Physical Science	Life, Physical, and Astronomical Science Studies	116
	Computer Science	Computers, Technology, IoT, Robotics and Coding Studies	117
	Transportation Science/Technology	Aviation, Shipping, Railways and Vehicular Studies	118

### Actionable recommendations

The seven points CCSP actionable recommendations are:

1. Policy Integration
  ○ Incorporate CCSP into the national education policy framework as a structured pathway linking pre-tertiary education to career opportunities.
  ○ Establish guidelines for curriculum alignment with CCSP to ensure relevance to both local and global labour markets.
2. Curriculum Development
  ○ Redesign curricula to embed CCSP options across junior and senior high schools, allowing students to select career-oriented subjects aligned with their interests and skills.
  ○ Regularly review and update curricula in consultation with industry stakeholders, higher education institutions, and professional bodies.
3. Capacity Building
  ○ Train teachers and administrators on CCSP implementation, career guidance, and inclusive pedagogical practices.
  ○ Establish partnerships with industries and vocational institutions to provide mentorship, internships, and practical training opportunities.
4. Equity and Inclusivity
  ○ Ensure CCSP is accessible to marginalized groups, including rural students, girls, and learners with disabilities, through targeted support schemes (scholarships, assistive technologies, outreach).
  ○ Develop monitoring tools to track participation and outcomes across different demographics.
5. Institutional Collaboration
  ○ Strengthen coordination between the Ministry of Education (MoE), Ghana Education Service (GES), and the private sector to support the rollout of CCSP.
  ○ Create a national CCSP advisory board comprising educators, policymakers, parents, and industry representatives.
6. Monitoring and Evaluation
  ○ Establish clear performance indicators (e.g., enrollment in CCSP tracks, transition rates to higher education or employment, student satisfaction).
  ○ Conduct periodic impact assessments to refine and improve the programme.
7. Awareness and Advocacy
  ○ Launch national awareness campaigns to sensitize parents, students, and communities about the benefits of CCSP.
  ○ Promote CCSP success stories to build trust and encourage wider adoption.

## Results and Discussions

Step 1: Problem Identification and General Needs Assessment

The three main problems identified are:

○ Young people are learning only at half capacity.
○ Quality of Education is poor.
○ Courses Offered for studies are limited.

Step 2: Targeted Needs Assessment

The Targeted Need Assessment focused on:

○ Courses and Curricula development

Step 3: Goals and Objectives

The Goals and Objectives have two tiers:

Stakeholders (Key Policy makers)

○ Adoption of CCSP to better serve the interest of the pre-tertiary students.
○ Provide the needed funding, resources and infrastructure.

Key partners (Pre-tertiary students)

○ Acquire the relevant basic educational knowledge and skills captured in the various curriculums and syllables.
○ Study the approved courses and prepare to take the necessary examinations and assessment for promotions and for the award of certificate.

Step 4: Educational Strategies

Educational strategies reviewed existing policies and promote new and improved policies. Here are three strategies:

○ Effective strategies vary based on level of education, the subjects, age group, and learning objectives.
○ Active Learning that allows students to actively participate in the learning process through discussions, problem-solving, and hands-on activities to meet the diverse needs of students. Students learn by asking questions and to integrate digital tools to solving problems using technology that enhance learning.
○ Social and Emotional Learning (SEL) focused on developing students’ emotional intelligence and interpersonal skills, and Project-Based Learning (PBL), where Students work on real-world projects over an extended period.

Step 5: Implementation

The implementation of CCSP require extensive works on the part of policy makers to enact the necessary policies and adoption.

○ CCSP adoption and implementation
○ Funding and promotion of CCSP

Step 6: Evaluation and Feedback

Assessment, Evaluation and Feedback of CCSP

○ Assess the progress and performance
○ Provide responses for improvement or recognition
○ Systematic process to measure the quality, effectiveness, or performance
○ Assess examination and assessment body, teaching and supervisory system, student and pupils’ evaluations, teaching materials and learning outcomes.
○ Feedback from the stakeholders; Examination and assessment bodies, Teaching and supervisory system, Student and pupils on the educational and curriculum reforms.
○ Feedback that communicates the evaluation results and suggests actions to improve or sustain performance.

## Agricultural Science: Farming, Fisheries, Forestry, Poultry, Animal Husbandry, and Food Production Studies

Agricultural Science Studies focus on basic educational knowledge of scientific principles and best agriculture practices, including traditional means of Farming, Forestry, Fishing. Agricultural Science include related food production, storage, marketing, resource management, and environmental sustainability. The study provides basics knowledge to integrates Crop Science, Animal Science, Soil Science, Agricultural Economics, Horticulture, Agricultural Engineering and Food Science.

### Agricultural Careers and Careers Requirements

Agricultural workers help to provide one of the main basic human needs, which is food. They work to produce all the food that we eat, and also some produce the materials that are used to make clothes, which also form part of the main basic needs of mankind. Careers in the Agriculture is listed in Table 2. Requirements: Agricultural Extension Officers have degrees or diplomas from Agricultural Colleges or a bachelor’s degree from a university. Mechanization and irrigation produced food on a larger scale. In the rural areas, most farmers and fishermen do not have formal education.

**Table 2.** Agriculture Science - Farming, Fisheries, Forestry and Food Production Careers.

Agricultural and Food Science Engineer	Biomass Plant Technician	Irrigation Specialist
Agricultural and Food Science Technician	Botanist	Landscape Agronomist
Agricultural Crop Farm Manager	Faller	Logging Worker
Agricultural Engineer	Farm Worker	Nursery Manager
Agricultural Equipment Operator	Farmer	Organic Farming Specialist
Agricultural Extension Officer	Farmers and Rancher	Pest Control Advisor
Agricultural Extension Officer and Inspector	Fish and Game Warden	Plant Pathologist
Agricultural Inspector	Fish Farmer	Precision Agriculture Specialist
Agricultural Scientist	Fisheries Biologist	Rangeland Manager
Agricultural Technician	Fisheries Manager	Silviculturist
Agriculturist	Fishing Crew	Soil and Water Conservationist
Agronomist	Floriculturist	Soil Scientist
Animal Breeder	Forest Conservationist	Sustainable Agriculture Consultant
Animal Farm Scientist	Forest Engineer	Timber Harvester
Animal Farmworker	Forest Ranger	Tree Surgeon (Arborist)
Beekeeper	Forest Technician	Veterinary Technician (Agriculture)
Biofuels Processing Technician	Forester	Wildlife Biologist (Agriculture and Forestry)
Dairy Farm Manager	Greenhouse Manager	Wood Technologist
Environmental Consultant (Agriculture)	Horticulturist	

### Administration - Administration, Secretarial and Office Support Studies

The Commercial Course offers learners basic educational knowledge in Administration, Secretarial and Office Support Studies, that equip students with the skills and experience required, to manage administrative tasks and support organizational operations effectively. This area of study is essential for the roles in business, government, education, healthcare, and other sectors.

### Administration, Secretarial, and Office Support Careers and Career Requirements

Organizations such as industries, businesses, the private sector, and other government agencies require workers in offices and administrative sectors, to ensure the efficient and smooth running of such organizations. Table 3 shows a list of Administration, and Office Support careers. Requirements: Most administrative and office support workers have degree from universities, or Diploma from higher school education. Other employers prefer college, vocational or secretarial and high schools job seekers.

**Table 3.** Commercial - Administration, Secretarial, and Office Support Careers.

Accounts Clerk	Data Entry Clerk	Marketing Manager
Administrative Assistant	Data Entry Operator	Medical Secretary
Administrative Coordinator	Data Entry Specialist	Office Coordinator
Administrative Manager	Database Administrator	Office Manager
Administrative Officer	Executive Assistant	Payroll Clerk
Administrator	File Clerk	Personal Assistant
Auditor	Financial Analyst	Postal Service Clerk
Bank Teller	Financial Manager	Project Administrator
Banker	Front Desk Clerk	Receiving Clerk
Bilingual Secretaries	General Office Clerk	Receptionist
Billing Clerk	Human Resources Assistant	Records Manager
Bookkeeping Clerk	Human Resources Coordinator	Scheduling Coordinator
Brokerage Clerk	Human Resources Manager	Secretary
Budget Analyst	Legal Secretary	Shipping Clerk
Cashier	Mail Carrier	Telephone Operator
Client Relations Specialist	Mail Clerk	Timekeeping Clerk
Customer Service Representative	Market Research Analyst	Typist

### Business and Finance - Business, Accounting, Banking, and Financial Studies

The Business, Accounting, Banking, and Financial Studies, provide the basic educational knowledge and training of a comprehensive field that covers various aspects of commerce, finance, and economic transactions. Sales and process of promoting and selling goods or services. Business activity of making, buying, or selling goods or services for profit. Banking the business conducted or services offered by a bank, including holding money for savings and checking accounts or issuing loans. Financial studies of how individuals, businesses, and organizations manage and use financial resources. This field is vital for understanding the dynamics of how businesses operate, how sales drive growth, and how financial systems underpin the economy, providing a foundation for careers in various industries.

### Careers in Business, Accounting, Banking, and Financial Services, Career Requirements

Workers in the areas of sales, service, Banking and finance inform people about products or services and persuade them to buy. Most sales workers sell their products in the markets and in shops and super markets. There are other sales representatives ready to answer questions about a product or to demonstrate how a product works. Careers in Sales, Business, Banking, and Finance options in listed in Table 4. Requirements: Companies that employ sales workers prefer to have at least high school education. Usually, they receive on-the-job training. Insurance sales agents, stockbrokers and other financial services employ people with a degree or college education. Wholesale, retail and manufacturing sale representatives prefer to have people with college or university degree.

**Table 4.** Business and Finance - Business, Accounting, Banking and Financial Careers.

Account Executive	Financial Planner	Telemarketer
Actuary	Insurance Sales Agent	Treasury Analyst
Advertising Sales Agent	Investment Banker	Wholesaler
Banker	Manufacturer’s Representative	Budget Analyst
Branch Manager	Marketing Manager	Accountant
Business Analyst	Operations Manager	Loan Officer
Business Development Manager	Real Estate Agent	Credit Analyst
Entrepreneur	Retail Banker	Financial Manager
Commercial Banker	Retail Sales Associate	Tax Preparer
Consultant	Risk Manager	Claims Adjuster
Data Scientist	Sales Manager	Insurance Underwriter
E-Commerce Specialist	Sales Representative	Financial Quantitative Analyst
Financial Analyst	Salesforce Administrator	Financial Risk Specialist
Businessman/Businesswoman	Stockbroker	

### Management - Management, Leadership, Politics, and Authority Studies

The Management, Leadership, Politics, and Authority Studies, provide the basic educational knowledge which delve into the principles and practices of guiding organizations, influencing political systems, and exercising power and authority. These fields are crucial for understanding how societies are organized, decisions are made, and leadership is exercised across various sectors. Management studies cover business, organizational behavior, financial management. Leadership ensures ethics, local and global politics, and international relations. Authority studies include sociology of power, legal authority and public administration. This course is vital for equipping individuals with the knowledge and skills to lead organizations, influence public policy, and understand the complex dynamics of power in society.

### Management, Leadership, Politics, and Authority Careers and Career Requirements

Management, Leadership, Politics, and Authority work force, called managers are the leaders of businesses and organizations. All businesses and organizations need managers to plan and administer activities and policies and to train and supervise other employees. Some managers are elected officials; they develop policies and laws and direct activities. The levels of management are different with different amount of authority and responsibility. Upper managers and chief executive officers, president and vice president have the most authority. Table 5 provide an option of careers in the Management, Leadership, Politics, and Authority. Requirements: Most managers, elected officials and management analysts are college graduates and many have advance degrees. Some organizations offer formal training programmes for their managers.

**Table 5.** Management - Management, Leadership, Politics, and Authority Careers.

Accountant	Director of Operations	Plant Foreman
Ambassador	Economic Development Director	Police Chief
Campaign Manager	Education Administrator	Policy Advisor
Center Manager	Executive Director	Political Analyst
Chief Executive Officer (CEO)	Foreman/Forewoman	Politician
Chief Financial Officer (CFO)	General Manager	President (of organizations or countries)
Chief Information Officer (CIO)	Governor	Press Secretary
Chief Marketing Officer (CMO)	Human Resources Manager	Product Manager
Chief Operating Officer (COO)	Immigration Officer	Project Manager
Chief Technology Officer (CTO)	Judge	Public Health Administrator
City Manager	Legislative Assistant	Public Relations Manager
City Planner	Manager	Regulatory Officer
Community Service Manager	Mayor	School Superintendent
Consultant	Military Officer	Social and Community Service Manager
County Administrator	Nonprofit Manager	State Representative
Customs and Border Protection Officer	Operations Manager	Supervisory Manager
Diplomat	Parliamentarian	

### Technical/Vocational - Technical, Construction, Repairs, and Maintenance, Fashion and Catering Studies

The Technical/Vocational - Technical, Construction, Repairs, and Maintenance Fashion and Catering Studies (TVET), provide basic educational knowledge that focuses on basics of design, building, fitting, mechanics, technicians and other specialized sectors such as Fashion, Clothing, and Catering Services. For effective working of automobiles, electricals, electronics, structures, infrastructure, and systems, these workforces are inevitable. These fields are essential for creating and maintaining the built environment, ensuring safety and sustainability. Construction, Maintenance, Repairs and Technical skills is prerequisite as Interdisciplinary Civil Engineering, Architecture, Environmental Engineering and Urban Planning and many others, to ensure that infrastructure meets current and future needs.

### Careers in Technical, Construction, Maintenance, and Repairs, and Career Requirements

Most workers in the field of construction help in the building of houses, roads, bridges, culverts, shops and other commercial building. Maintenance and repair workers help to maintain machinery and other properties and fix them when they are faulty or not working properly. Construction, Maintenance, and Repairs careers shown in Table 6 offers option in this category. Requirements: Most workers in this field of careers have a minimum of JHS or SHS. Others learn or attend either a technical or a vocational school, whiles others have degrees from university. Mostly the fitters learn their skills through on-the-job training.

**Table 6.** Technical/Vocational - Technical, Construction, Repairs and Maintenance, Fashion and Catering Careers.

Assembler and Fabricator	Roofer	Insulation Installer
Automatic Teller Machine Attendant	Tile and Marble Setter	Landscaper & Groundskeeper
Automotive Body Repairer	Air Conditioning Technician	Machinery Maintenance Worker
Automotive Engineer	Automatic Teller Machine (ATM) Attendant	Marine Mechanic
Automotive Engineering Technician	Boilermaker	Mason (Brick, Stone, Cement)
Automotive Glass Installer & Repairer	Bridge Construction Worker	Metal Fabricator
Automotive Master Mechanic	Cabinetmaker	Millwright
Automotive Mechanic	Carpet Installer	Oil and Gas Driller
Automotive Specialty Technician	Ceiling Tile Installer	Pipefitter
Automotive Technician and Mechanic	Cement Mason and Concrete Finisher	Plasterer
Auxiliary Power Equipment Operator	Construction Equipment Operator	Power Plant Maintenance Technician
Aviation Inspector	Construction Foreman	Railroad Maintenance Technician
Avionics Technician	Construction Laborer	Refrigeration Mechanic
Bricklayer	Crane Operator	Rigger
Carpenter	Demolition Worker	Scaffolder
Carper Installer	Diesel Mechanic	Sheet Metal Worker
Concrete Mason	Dock and Port Maintenance Technician	Shipbuilder and Repair Technician
Electrician	Drywall Finisher and Installer	Solar Panel Installer
Elevator Installer and Repairer	Equipment Operator (Heavy Machinery)	Stonemason
Exterminator	Excavator Operator	Structural Iron and Steel Worker
Fitter	Exterminator (Pest Control Technician)	Tire Technician
Gas Appliance Repairer	Flooring Installer	Tool and Die Maker
Gearbox Specialist	Furniture Finisher	Tower Climber & Maintenance Technician
Generator Mechanic	Glazier	Upholsterer
Janitor	Handyman	Vehicle Maintenance Supervisor
Locksmith	Hazardous Materials Removal Worker	Wastewater Treatment Operator
Maintenance & Repair Worker	Heavy Equipment Mechanic	Welder
Construction Electrician	Heating, Ventilation, and AC Technician	Window Installer
Motorcycle Mechanic	Industrial Machinery Mechanic	Woodworker
Painter	Caterer	Wind Turbine Technician
Plumber	Fashion Designer	Dress Maker

### Industrial Technology - Production, Manufacturing and Industrial Studies

The Industrial Technology - Production, Manufacturing, and Industrial Studies, provide the basic educational knowledge of broad field that encompasses various aspects of creating goods and managing industrial processes. Production teaches the process of transforming raw materials into finished goods through various methods and processes. Manufacturing provides steps through which raw materials are transformed into a final product. Industrial Studies is an interdisciplinary field that examines industrial processes, systems, and organizations to improve efficiency, productivity, and sustainability. Technological Integration provide the use of control systems for operating equipment. Applications of Health and Safety in addition to Research and Development (R&D). This field is dynamic and continuously evolving with advancements in technology and shifts in global markets, making it a vital area of study for improving industrial efficiency and innovation.

### Careers in Production, Manufacturing, and Industry, and Career Requirements

Workers in this category are involved in making or preparing goods either by hand or machine. These products range from simple wooden objects such as tables and chairs to complex computer parts. Some production workers are involved in food processing. Production, Manufacturing, and Industrial careers options is provided in Table 7. Requirements: Most workers in this group have to study at college, technical or vocational school. Others pursue apprenticeship programs. In other area, JHS or SHS level is required, while in other sectors formal education and Bachelor’s or Master’s degree is required.

**Table 7.** Industrial Technology - Industrial, Manufacturing, and Production Careers.

AI and Data Analyst	Logistics Coordinator	Chemical Plant Operator
Assembler	Machine Operator	Food Processing Operator
Assembly Line Worker	Machinist	Metal and Plastic Machine Worker
Baker	Manufacturing Engineer	Ophthalmic Laboratory Technician
Bindery Worker	Manufacturing Technician	Painting and Coating Worker
Butcher	Meat Dresser	Power Plant Operator
CNC Machinist	Millwright	Semiconductor Processor
Desktop Publisher	Mine Worker	Sewer and Tailor
Detailer	Precision Assembler	Slaughterer and Meat Packer
Dressmaker	Prepress Worker	Textile Worker
Industrial Electrician	Printing Press Operator	Tool and Die Maker
Environmental Engineer	Procurement Specialist	Welder
Industrial Automation Specialist	Production Manager	Woodworker
Industrial Designer	Quality Control Specialist	Quality Engineer
Industrial Hygienist	Robotics Technician	Process Engineer
Inventory Manager	Safety Officer	Lithographer
Jeweler	Boilermaker	

### Home Science - Home Economic, and Personal Services Studies

The Home Science - Personal Services, and Home Economics Studies, provide the basic educational knowledge that focuses on skills and knowledge related to individual well-being, family management, and consumer services. These fields prepare individuals for careers in service-oriented industries and equip them with practical skills for managing personal and household needs. Personal Services include Cosmetology and Beauty, Fitness and Personal Training, Health and Wellness. Home Economics covers Family and Consumer Sciences, Textiles and Clothing, and Home Management, Food and Nutrition. Personal Services and Home Economics Studies are integral for preparing individuals to provide essential services that enhance quality of life, foster healthy living, and support personal and family well-being.

### Careers in Personal Services, and Home Economic, and Career Requirements

Some of the workers in this category require special skills to perform personal task for people. Many personal services are the type of services people can do themselves but for one reason or the other they prefer that specialist in these areas perform these tasks. Careers in Personal Services, and Home Economics in listed in Table 8. Requirements: Most workers in this field require formal education or training. However, at least Junior school level may be required, other workers receive on the job training, some may have to attend special training and have degree.

**Table 8.** Home Science - Home Economics and Personal Services Careers.

Animal Caretaker	Hairstylist	Family and Consumer Sciences Educator
Animal Trainer	Home Care Aide	Home Economist
Barber	Home Health Aide	Massage Therapist
Beautician	Janitor and Cleaner	Personal Shopper
Bridal Consultant	Makeup Artist	Tailor/Dressmaker
Clergy	Manicurist	Laundry and Dry-Cleaning Worker
Concierge	Marriage and Family Therapist	Social and Community Service Manager
Cosmetologist	Nanny	Event Planner
Embalmer	Pedicurist	Butler
Fitness Trainer	Child Care Provider	Sommelier
Flight Attendant	Dietitian/Nutritionist	Tour Guide
Floral Designer	Interior Designer	Personal Assistant
Funeral Director	Culinary Professional	Life Coach
Hairdresser		

### Hospitality and Tourism - Hospitality, Hotelier, Travel and Tourism Studies

The Hospitality and Tourism - Hospitality, Hotelier, Travel and Tourism Studies, is to provide the basic educational knowledge of Hospitality and tourism industries, such as hotels, restaurants, travel agencies, event planning and tourism management. It provides the framework of the importance of customers and perspective of global tourism and travel trend and destination management.

### Careers in Hospitality, and Tourism, Hotelier, and Travel and Tour, Career Requirements

People who work in the field of tourism and hospitality provide services that help people to enjoy their vacations and leisure times. Some hospitality services plan their activities, which include recreational services. Other makes the guest happy while they enjoy live programs and services. Table 9 is the list of careers in the Hospitality, and Tourism industries. Requirements: At least high school education is required in most tourism and hospitality services. Travel and tour services also prefer people with vocational education, technical, college, polytechnic or university graduate.

**Table 9.** Hospitality and Tourism - Hospitality, Hotelier, Travel and Tourism Careers.

Accommodation Manager	Exhibition Coordinator	Restaurant Host/Hostess
Adventure Tourism Guide	Flight Attendant	Restaurant Manager
Airline Customer Service Agent	Food and Beverage Director	Revenue Manager
Airline Reservation Agent	Food Critic	Ski Resort Manager
Airport Duty Manager	Food Safety Inspector	Sommelier (Wine Expert)
Airport Manager	Food Stylist	Spa Manager
Baker	Front Desk Agent	Sports Tourism Manager
Banquet Manager	Front Desk Clerk	Sustainable Tourism Specialist
Bartender	Guest Experience Manager	Theme Park Manager
Bed and Breakfast Owner	Hospitality Consultant	Tour Guide
Bellhop/Bell Attendant	Hospitality Trainer	Tour Operator
Butler	Hotel General Manager	Tourism Marketing Manager
Caterer	Recreational Manager	Tourism Research Analyst
Catering Manager	Hotel Manager	Travel Agent
Chef	Hotel Receptionist	Travel Blogger
Concierge	Hotel Reservation Agent	Travel Consultant
Conference and Event Coordinator	Hotelier	Travel Influencer
Cook	Housekeeper	Travel Photographer
Corporate Travel Manager	Housekeeping Manager	Travel Show Host
Cruise Director	Lodge Manager	Vacation Rental Manager
Cruise Line Manager	Luxury Travel Advisor	Waiter and Waitress
Cruise Ship Casino Dealer	Night Auditor (Hotel Accounting)	Waiter/Waitress
Cruise Ship Entertainer	Park Ranger (Eco-Tourism Sector)	Wellness Retreat Coordinator
Cruise Ship Staff	Personal Chef (Luxury Hospitality)	Wildlife Tour Operator
Culinary Instructor	Recreation Manager	Wine Tour Guide
Destination Manager	Resort Activities Coordinator	Yacht Crew Member
Eco Tourism Guide	Resort Manager	Yacht Steward/Stewardess
Event Planner	Restaurant Host and Hostess	

### Audio Visual, and Physical Arts: Audio Visual, Performing and Literary Arts, Sports and Music - Sports, and Design Studies

The Sports, Music and Entertainment Studies (SMES), provide the basic educational knowledge of interdisciplinary field which focuses on preparing students for careers in sports, music and entertainment industries. The course provides combine creative technical and artistic skills. Sports management, athlete representation, music production, concert and event promotion, Film, Radio and Television production.

### Careers in Audio Visual, Performing and Literary Arts, Sports and Music - Sports, and Design Studies, and Career Requirements

Millions of people are actively involved in sporting events and enjoy the excitement of competition. Entertainment activities amuse many audiences. Others, like beauty contests and concert performances, are also enjoyed by thousands of people. Some workers specialized in certain sporting and athletic activities. Sports, Music, and Entertainment career options are provided in Table 10. Requirements: For many careers in the sports and entertainment group, there are no specific educational requirements before one cold engage in such activities. Most of the people engaged in sports or entertainment have natural talent and they practice or train for hours in a day. Many athletes earn degrees while they compete in college athletics programs. Officials of sports were in most cases sports personnel and may specialize in coaching or as trainers. Other attends colleges or earns degree from university.

**Table 10.** Sports, Music - Sports, Music, Acting, and Entertainment Careers.

Acoustician	Cyclist	Goalkeeper
Actor/Actress	Dancer	Music Director
Amusement Attendant	Director (Television, and Radio)	Screenwriter
Athlete	Disc Jockey	Set Designer
Balloonist	Entertainer	Sound Engineer
Bandleader/Bandmaster	Fashion Coordinator	Sports Announcer
Bandsman	Fashion Designer	Sports Official/Referee
Baseball Player	Fashion Editor	Stunt Performer
Basketball Player	Fashion Photographer	Talent Agent
Choreographer	Fashion Writer/Artist	Theater Manager
Coach	Film Director	Video Game Designer
Comedian	Film Laboratory Technician	Voice-over Artist
Composer	Fitness Trainer	Wardrobe Stylist
Conductor	Footballer	Wedding Planner
Yoga Instructor		

### Multimedia Arts: Radio and Television, Media, Communication, Journalism Studies

The Multimedia Arts – Radio and Television, Media, Communication, and Journalism Studies, provides basic educational knowledge that covers a diverse range of disciplines focused on creative, analysis, and interpretation of audio visual and media content. The Art studies involve exploration of Visual Arts, Performing Arts and Literary Arts. It focuses on both practical skills and creativity which require theoretical and practical tutelage. Media studies examine production, distribution, and consumption of media content, such as film, television, radio, and online platforms. Communication studies delve into the ways people share information through various channels, including Journalism, and digital communication. Design studies focus on the principles and practices of creativity, fashion, graphic design, industrial design, and interior design.

### Art, Media, Communication, and Design Careers, and Career Requirements

Occupations in the areas of art, media, communications, and design, express their thought, feeling, ideas and current affairs in their work, provide beauty to the lives of their customers. Others express their ideas through information processing. An option of careers in Art, Media, Communication, and Design has been provided in Table 11. Requirements: A greater number of artists have natural artistic abilities. Some artists have to undergo training, while some have no formal training. However, others have a degree in fine arts from a university, college, or school of art. Most designers have diploma from polytechnics. In the field of communication, a bachelor’s degree or master’s degree is a requirement.

**Table 11.** General Arts - Art, Media, Communication, and Design Careers.

Anchorman	Creative Director	Music Director
Anchorwoman	Curator	Musician
Animator	Dancer	Musicologist
Art Director	Design Director	Photographer
Art Therapist	Designer	Photojournalist
Artist	Digital Media Specialist	Podcast Producer
Audio Engineer	Disc Jockey (DJ)	Production Designer
Author	Editor	Proofreader
Blogger	Event Planner	Public Relations Officer
Brand Identity Developer	Fashion Designer	Public Relations Specialist
Broadcast News Analyst	Film Director	Radio Announcer
Broadcast Technician	Fine Artist	Reporter
Broadcaster	Floral Designer	Set Designer
Calligrapher	Game Designer	Social Media Manager
Camera operator	Graphic Designer	Sound Designer
Cartoonist	Illustrator	Technical Writer
Cinematographer	Industrial Designer	Television Anchor
Colorist	Interior Designer	Television Producer
Columnist	Jewelry Designer	User Experience (UX) Designer
Commercial Artist	Journalist	Video Editor
Communications Manager	Lighting Technician	Videographer
Composer	Makeup Artist	Visual Effects (VFX) Artist
Concept Artist	Media Buyer	Voice-over Artist
Content Strategist	Media Planner	Web Designer
Copywriter	Motion Graphics Designer	Writer
Costume Attendant	Multimedia Artist	

### Law and Legal Arts - Law, Legal Systems, and Human Rights Studies

The Law and Legal Systems - Law, Legal Systems, and Human Rights Studies, is to provide basic educational knowledge involve in the exploration of legal principles, institutions, and practices that govern societies. This field encompasses analysis of laws, the role of legal institutions, constitutions and the impact of legal systems on individuals and communities. It is essential for understanding the frameworks that maintain order, protect rights, and resolve disputes. The Course provides foundation of law, civil law, criminal law, public law, international law, corporate and commercial law, dispute resolution, legal systems and institutions. The basics of Law and Legal practice are critical for training legal professionals, shaping public policy, and understanding the legal foundations that support justice and governance in society.

### Careers in Law, Legal Systems, and Human Rights, and Career Requirements

Workers in these categories help people to protect and preserve their rights and freedom. Law is one of the basic social institutions without which no society could exist if people could do just what they pleased. Within every society each person has some obligation towards one another. Table 12 provides the list of careers in Law, and Legal Practice. Requirements: Most lawyers have college education and degree from a Law schools and universities. Most judges have been lawyers. Workers in the paralegals usually have an associate or a bachelor’s degree, and may have specialized legal training.

**Table 12.** Law and Legal System - Law, Legal System and Human Rights Practice Careers.

Adjudicator	E-Discovery Specialist	Legal Operations Manager
Administrative Law Judge	Hearing Officer	Legal Secretary
Arbitrator	Human Rights Lawyer	Legal Technology Specialist
Attorney	Immigration Lawyer	Legislative Counsel
Attorney General	Judge	Legislator
Bailiff	Judicial Law Clerk	Magistrate
Civil Rights Advocate	Law Clerk	Magistrate Judge
Compliance Officer	Law Librarian	Mediator
Conciliator	Lawyer	Paralegal
Contract Manager	Legal Assistant	Professional Support Lawyer
Corporate Counsel	Legal Consultant	Prosecutor
Court Clerk	Legal Contractor	Title Examiner, Abstractor, and Searcher
Court Interpreter	Legal Operations Analyst	Victim Advocate
Court Reporter		

### Education - Education, Training Teaching and Learning, and Faculty Studies

The Education – Education, Training, Teaching, and Learning studies to offer basic educational knowledge of teaching, training and education studies as academic and professional field focused on the theory and practice of facilitating learning and skill development, as pre-requisite entry requirement to teacher training colleges and universities.

### Careers in Teaching, Education, and Training, and Career Requirements

Workers in this professional group offer systematic information to students based on subjects. They teach many kinds of skills and cultural values to students of all ages. A variety of methods of teaching are employed, including practical, laboratory, workshops, presentations, demonstrations, excursions, research, the use of computers and information technology, and project presentations. While others practice a Dual Education System. Career options in Teaching, Education, and Training are provided in Table 13. Requirements: Almost all teachers need a bachelor’s degree. Others are required to complete a professional teacher training program before they can teach. Some pre-school teachers and teacher’s aides do not need a degree. Many high school teachers and most college and university faculty have advanced degrees. Those teaching in the universities have a doctorate degree others require a minimum of a master’s degree.

**Table 13.** Education - Education, Training, Teaching and Learning Careers.

Adapted Physical Education Specialist	Education Teacher (Postsecondary)	Library Science Teacher (Postsecondary)
Adult Literacy and Remedial Education Teacher	Elementary School Teacher	Life, and Physical Science Studies Teacher
Administration, and Office Support Teacher	Engineering Science, and Mathematics Studies Teacher	Management, Leadership, Politics, and Authority Studies Teacher
Agricultural Science Teacher (Postsecondary)	Engineering Teacher (Postsecondary)	Mathematical Science Teacher (Postsecondary)
Anthropology and Archaeology Teacher (Postsecondary)	English Language and Literature Teacher	Montessori Teacher
Architectural Teacher (Postsecondary)	Environmental Science Teacher (Postsecondary)	Music Instructor (Private & Institutional)
Area, Ethnic, and Cultural Studies Teacher	Forestry and Conservation Science Teacher	Personal Services, and Home Economics Studies Teacher
Art, Drama, and Music Teacher	Geography Teacher	Physical Education Teacher
Art, Media, Communication, and Design Teacher	Health Science Studies Teacher	Physics Teacher (Postsecondary)
	Head Teacher	Preschool Teacher
Atmospheric, Earth, Marine, and Space Science Teacher	Headmaster/Headmistress	Principal/School Administrator
Biological Science Teacher (Postsecondary)	Health Specialties Teacher (Postsecondary)	Production, Manufacturing, and Industrial Studies Teacher
Business Teacher (Postsecondary)	History Teacher (Postsecondary)	Psychology Teacher (Postsecondary)
Chemistry Teacher (Postsecondary)	Home Economics Teacher (Postsecondary)	Sales, Commerce, Banking, and Financial Studies Teacher
College and University Faculty	Hospitality, and Tourism Studies Teacher	Science Teacher (General)
Communications Teacher (Postsecondary)	Instructional Coordinator	Secondary School Teacher (SHS)
Community, Civil Protection, and Social Studies Teacher	Junior High School Teacher (JHS)	Special Education Teacher
Computer Science Teacher (Postsecondary)	Kindergarten Teacher	Sports, Music, and Entertainment Studies Teacher
Construction, Maintenance, Repairs, and Technical Studies Teacher	Law, and Legal Systems Studies Teacher	Teacher Trainer/Educational Consultant
Continuing Education Teacher	Law Teacher (Postsecondary)	Teaching Assistant
Criminal Justice/Law Enforcement Teacher	Transportation, and Facilities Studies Teacher	Teaching, Education, and Training Studies Teacher
Early Childhood Education Teacher	Tutor/Private Educator	Technical and Vocational Education Teacher
Economics Teacher (Postsecondary)		Technology, IoT, Computer, and Coding Studies Teacher

### Social Science - Humanities, Community, Civil Protection, and Social Studies

The Social Science - Humanities, Community, Civil Protection, and Social Studies, provide basic educational knowledge of societal structures, the role of community engagement, and strategies for ensuring public safety and well-being. The study is essential for understanding and improving the quality of life in various communities through effective governance, social support systems, emergency management, Police, Military, and Para-Military Services for defense and protection.

### Humanities, Community, Civil Protection, and Social Services Careers, and Career Requirements

People engaged in community and social services provide assistance to society, in order to improve the quality of life in their neighborhood and in the societies in which they find themselves. People depend on these workers to help them individually or as a group. They also help families solve their problems. Counselors in particular help people identify their problem and find solutions. Other social workers offer assistance in emergency cases. Careers in Community, Civil Protection, and Social Services has been provided in Table 14. Requirements: At least a High School level of education is the minimum requirement for all branches of the forces such as the Army, Air-force, Police, Fire, Prison, Customs, and Immigration services and other voluntary services. A diploma or university degree may be preferred in some cases or a professional qualification. Most members of the Clergy attend seminaries, other field within the community and social services require degree from a university.

**Table 14.** Social Science – Humanities, Community, Civil Protection, and Social Services Careers.

Air Force Personnel	Family and Youth Services Worker	Police Chaplain
Artillery Personnel	Fire Fighter	Police Detective
Bailiff	Fire Inspector	Police Officer
Border Patrol Agent	Fire Inspector and Investigator	Prison Chaplain
Case Manager	Forensic Social Worker	Prison Officer
Child and Family Social Worker	Forest Fire Fighter	Probation Officer
Child Protection Officer	Foster Care Caseworker	Public Defender (Social Justice Law)
Child Welfare Specialist	Government Social Policy Analyst	Public Health Advocate
Clergy	Homeless Shelter Coordinator	Public Safety Officer
Community Development Officer	Human Rights Advocate	Rehabilitation Counselor
Community Health Worker	Human Rights Lawyer	Search and Rescue Technician
Community Outreach Coordinator	Human Services Administrator	Security Consultant (Community Safety)
Community Service Manager	Infantry Soldier	Sexual Assault Crisis Counselor
Correctional Counselor	Juvenile Caseworker	Shelter Worker (Domestic Violence, Homeless)
Correctional Treatment Specialist	Juvenile Probation Officer	Social Policy Analyst
Counselor (Substance Abuse, Mental Health, Family)	Librarian	Social Services Coordinator
Crime Scene Investigator (CSI)	Marriage and Family Therapist	Social Worker
Criminal Justice Advocate	Mediator	Suicide Prevention Counselor
Curator	Mental Health Advocate	Support Group Facilitator
Custom Officer	Military Personnel	Victim Advocate
Detective & Criminal Investigator	Municipal Fire Fighter	Violence Prevention Specialist
Disaster Response Coordinator	Nonprofit Program Coordinator	Volunteer Coordinator (Community Services)
Domestic Violence Counselor	Paralegal (Community Legal Services)	Youth Caseworker
Drug and Alcohol Counselor	Parole Officer	Youth Counselor
Emergency Management Director		Youth Development Specialist

### Health Science - Health Care, Medicine, and Allied Health Studies

The Health Science - Health Care, Medicine, and Allied Health Studies, provide basics educational knowledge of scientific, social, and behavioral aspects of health care and wellness, focusing on the prevention, diagnosis, and treatment of diseases and the promotion of overall health. This interdisciplinary field of study integrates knowledge from biology, chemistry, medicine, psychology, and public health to improve individual and community health outcomes. HSS cover basics of Biomedical Sciences, Public Health, Clinical Medicine, Nursing and Allied Health Professions, Nutrition and Dietetics, Pharmacy and Pharmacology, Mental Health and Psychology, Health Informatics and basics herbal medicine. Health Science Studies are fundamental for advancing healthcare practices, improving patient outcomes, and promoting public health on a global scale.

### Careers in Health Care, Medicine, and Allied Health Services, and Career Requirements

Workers in this category provide a wide range of services, which help people live healthier and happier lives. They prevent, diagnose and treat illnesses. And in some case complex surgical operation or transplant could be performed. Table 15 provide several career options in the Health Science and Health care services. Requirements: Preparation towards health care careers can take many years of studies. In most cases a Bachelor’s degree has to be obtained before the actual medical training begins. In other areas a Bachelor’s, Master’s or Associate degrees has to be obtained. And most healthcare assistance may have to attend specialized institutions to acquire a degree or diploma.

**Table 15.** Health Science - Health Care, Medicine, and Allied Health Services Careers.

Acute Care Nurse	Health Information Technician	Pharmacist
Allergist and Immunologist	Immunologist	Physical Therapist
Anatomist	Laboratory Technician	Physician
Anesthesiologist	Magnetic Resonance Imaging Technologist	Physician Assistant
Anesthesiologist Assistant	Medical and Clinical Laboratory Technician	Physiotherapist
Anesthetist	Medical and Clinical Laboratory Technologist	Plastic Surgeon
Audiologist	Medical and Health Services Manager	Podiatrist
Biomedical Engineer	Medical and Public Health Social Worker	Psychiatric Nurse
Cardiologist	Medical Appliance Technician	Radiologist
Cardiovascular Technologist	Medical Assistant	Radiotherapist
Chemist	Medical Microbiologist	Registered Nurse
Chiropractor	Medical Officer	Speech-Language Pathologist
Clinical Neuropsychologist	Medical Practitioner	Surgeon
Clinical Nurse Specialist	Medical Record	Traditional Birth Attendant
Clinical Psychologist	Medical Records Technician	Urologist
Clinical Research Coordinator	Medical Scientist	Veterinarian
Community Health Worker	Medical Secretary	Ward Assistant and Ward Help
Critical Care Nurse	Medical Transcriptionist	Mental Nurse
Dental Assistant	Mental Health Counselor	Midwife
Dental Hygienist	Mental Nurse	Neurodiagnostic Technologist
Dental Laboratory Technician	Midwifery	Neurologist
Dentist	Neurodiagnostic Technologist	Neuron physiologist
Dermatologist	Neurologist	Neuropathology
Diagnostic Medical Sonographer	Neuron Physiologist	Neuropsychologist
Dietetic Technician	Neuropathology	Neurosurgeon
Dietitian	Neuropsychologist	Nuclear Medicine Physician
Dietitian and Nutritionist	Nuclear Medicine Physician	Nuclear Medicine Technologist
Echo Cardiographer	Nuclear Medicine Technologist	Nurse
Emergency Medical Technician	Obstetrician	Obstetrician
Endocrinologist	Ophthalmic Optician	Occupational Therapist
Endoscopy Technician	Orthopedist	Ophthalmic Optician
Epidemiologist	Pediatrician	Ophthalmologist
Etiologist	Physician	Opticians
Family and General Practitioner	Geriatrician	Optometrist
Gastro Entomologist	Gynecologist	Pharmacist
General Dentist		Physical Therapist

### Engineering Science - Engineering, Innovation and Mathematics Studies

The Engineering Science - Engineering, Innovation, and Mathematics Studies, provide the basic educational knowledge of a broad interdisciplinary field that integrates principles of engineering, scientific methods, and mathematical analysis to solve real-world complex technical problems by focusing on core concepts in physics, chemistry, materials science, and mathematics. Engineering Science focuses on applying scientific principles to real-world engineering problems. Bridge the gap between theoretical science and practical engineering applications such as Aerospace, Biological, Civil, Chemical, Computer, Environmental, Electrical and Electronics. Mathematics for Engineering provides the quantitative tools needed for problem-solving and system modeling.

### Engineering Science, Innovation and Mathematics Careers, Career Requirements

Professional in Engineering Science and Mathematics field like mechanical, electrical, civil, and chemical engineering have the ability, knowledge, experience and problem-solving skills. They develop analytical and critical thinking skills to tackle engineering challenges. Using Innovation and Research to supports technological advancements in emerging areas like renewable energy, robotics, and Artificial Intelligence (AI). Careers in Engineering and Mathematics is shown in Table 16, and offer alternative careers options. Requirements: Most engineers require basic strong foundation in Mathematics, Physics, Chemistry, Biology and Computer Science to pursue a degree in Engineering Science and Mathematics. Professionals in this field require Bachelor’s Degree (BEng/BSc), Master’s Degree (MEng/MSc), or PhD (Doctorate).

**Table 16.** Engineering Science - Engineering, Innovations and Mathematics Careers.

Acoustical Engineer	Construction Electrician	Meteorologist
Actuary	Control Systems Engineer	Mining Engineer
Aerospace Engineer	Control/Instrumentation Engineer	Molecular Biologist
Aerospace Technician	Cryptographer	Nanoengineer
Aircraft Mechanic	Cybersecurity Engineer	Network Engineer
Aircraft Systems Engineer	Data Analyst	Nuclear Engineer
Architect	Data Scientist	Oceanographer
Architectural Drafter	Design Engineer	Operations Research Analyst
	Draftsman	Optical Engineer
Architectural Engineer	Ecologist	Petroleum Engineer
Architectural Technician	Electrical Engineer	Pharmaceutical Scientist
Artificial Intelligence Engineer	Electrical Engineering Technologist	Photonics Engineer
Astrobiologist	Electrician	Physicist
Astronomer	Electromechanical Equipment Assembler	Planetary Scientist
Astrophysicist	Electronics Drafter	Plasma Physicist
Automation Engineer	Electronics Engineer	Process Engineer
Automobile Mechanic	Energy Systems Engineer	Quantum Computing Scientist
Automotive Engineer	Environmental Engineer	Quantum Physicist
Biochemical Engineer	Environmental Scientist	Remote Sensing Scientist
Biofuels Processing Technician	Epidemiologist	Renewable Energy Engineer
Biological Scientist	Forensic Scientist	Research Scientist
Biological Technician	Genetic Engineer	Robotics Engineer
Biomass Plant Technician	Geologist	Satellite Engineer
Biomechanics Engineer	Geophysicist	Seismologist
Biomedical Engineer	Industrial Engineer	Software Engineer
Biostatistician	Information Security Analyst	Solar Energy Engineer
Broadcast Technician	Instrumentation Engineer	Statistician
Cartographer	Machine Learning Engineer	Structural Engineer
Cartographer (Map Maker)	Manufacturing Engineer	Surveyor
Chemical Engineer	Marine Engineer	Systems Engineer
Chemical Technician	Materials Engineer	Telecommunications Engineer
Chemist	Mathematician	Theoretical Physicist
Civil Drafter	Mechanical Drafter	Transportation Engineer
Civil Engineer	Mechanical Engineer	Waste Management Engineer
Civil Engineering Technician	Mechatronics Engineer	Water Resources Engineer
Computer Engineer	Metallurgical Engineer	Wind Energy Engineer

### Life, Physical, and Astronomical Science Studies

The Life, Physical and Astronomical Science Studies, provide the basic educational knowledge of a wide range of disciplines that explore the natural world, from the molecular mechanisms of life, to the fundamental principles governing the universe. These fields are crucial for advancing scientific knowledge, technological innovation, and understanding the complexities of the environment and living organisms. Life Science Studies include Biology, Biochemistry, Botany, Zoology, Microbiology. While Physical Science Studies covers Physics, Chemistry, Earth Science and Astronomy. The foundational for scientific discovery and innovation, addressing global challenges, and improving the quality of life through a deeper understanding of natural phenomena.

### Life, Physical, and Astronomical Science Careers, and Career Requirements

Careers within these categories, mostly scientists, their discoveries and redevelopment help us to understand the functions of the world in which we live. There are areas where prevention is needed and where new development is required. Life, Physical, and Social Science careers in Table 17, offers option of career types in this category. Requirements: Most scientists have doctorate degree. A minimum of bachelor’s degree is required. In most cases these scientists and doctor of philosophy (PhD) undertake research with research assistance.

**Table 17.** Life and Physical - Life, and Physical Science Careers.

Agricultural Scientist	Environmental Economist	Forensic Scientist
Agronomist	Environmental Engineer	Hydrologist
Animal Scientist	Environmental Scientist	Marine Biologist
Anthropologist	Epidemiologist	Materials Scientist
Archaeologist	Geneticist	Meteorologist
Astronomer	Geochemist	Microbiologist
Astrophysicist	Geochronologist	Neuroscientist
Atmospheric Scientist	Geodetic Surveyor	Oceanographer
Bacteriologist	Geographer	Paleontologist
Biochemist	Geologist	Physicist
Biologist	Geomorphologist	Political Scientist
Biophysicist	Geophysicist	Psychologist
Biotechnologist	Geoscientist	Sociologist
Botanist	Biological Technician	Soil Scientist
Climatologist	Cartographer	Statistician
Conservation Scientist	Chemical Technician	Survey Researcher
Cosmologist	Chemist	Urban and Regional Planner
Economist	Ecologist	Zoologist
Environmental Compliance Inspector		

### Computer Science – Computer, Technology, IoT, Robotics and Coding Studies

The Computer Science – Computer, Technology, IoT, Robotics and coding Studies focus on the basic educational knowledge of understanding, designing and applying modern technological systems and programming languages of coding to solve real word problems. Study tools and systems such as Artificial Intelligence (AI), Cybersecurity, Data Science, Cloud Computing and Human-Computer Interaction (HCI).

### Careers in Computers, Technology, Internet-of-things, Computer, and Coding, and Career Requirements

Workers in this group use computer and technology to prepare or analyze a variety of complicated procedures. Business and organization cannot function efficiently without these professionals who design complicated items like the microprocessor clip, computers, automobile, aircraft and other complex projects. Careers in the Technology, Internet-of-things, Computer, and Coding in provided in Table 18. Requirements: Almost all the professionals in the fields of Computer Science and Technology, either have to complete their education at a technical institution, college, polytechnic or university. Most employers require a bachelor’s degree.

**Table 18.** Computer Science - Computer, Technology, IoT, and Coding Studies Careers.

Actuaries	Computer Systems Analyst	Information Security Analyst
AI Research Scientist	Computer Technician	IoT Architect
Artificial Intelligence Specialist	Computer/Office machine Technician	IoT Developer
Augmented Reality (AR) Developer	Cybersecurity Analyst	Machine Learning Engineer
Blockchain Developer	Data Analyst	Mobile Application Developer
Cloud Engineer	Data Scientist	Network Engineer
Cloud Solutions Architect	Database Administrator	Robotics Engineer
Computer Network Architect	Database Architect	Systems Analyst
Computer Programmer	DevOps Engineer	User Experience (UX) Designer
Computer Security Specialist	Embedded Systems Engineer	Virtual Reality (VR) Developer
Computer Software Engineer	Game Developer	Web Developer
Computer Support Specialist	Hardware Engineer	

### Transportation - Aviation, Shipping, Railways and Vehicular Studies

The Transportation - Aviation, Shipping, Railways and Vehicular Studies, provide basic educational knowledge in planning, design, management and optimization of transportation systems and facilities. It emphasizes sustainability, efficiency and safety. The transportation sector include; Air, Rail, Road, and Water transportation. The study provides knowledge acquisition in the systems and network of Aviation, Aircraft and Airport systems. It also includes; Maritime, Shipping, Harbour, Port freight and forward, import and export systems, railway and train service system, Heavy duty vehicles and light trucks, commercial Buses and private cars used to move people, goods and services efficiently and safely.

### Careers in Transportation - Aviation, Shipping, Railways and Vehicular Studies, and Career Requirements

People, goods and services moving from one place to another depend greatly on transport services. These services range from air, sea, rail and road transportation, worker in the field of transport help passengers and goods to move from one place to another. Transportation, and Facilities careers Table 19 provide an option of water, rail, road and air transportation. Requirements: At least all qualified drivers should have basic formal education and should possess valid driver’s license. Aircraft pilot should have bachelor’s degree. Many water transportation workers have professional training or bachelor’s degree from Maritime University or nautical college. Air transportation requires special training relating to aviation and airport systems.

**Table 19.** Transportation - Aviation, Shipping, Railways and Vehicular Careers.

Able Seaman	Aircraft Systems Assembler, Precision	Commercial Pilot
Agricultural Machinery Operator	Aircraftman	Construction Equipment Operator
Air Chief Marshal	Airfield Operations Specialist	Automotive Mechanic
Air Crew Member	Airframe-and-Power-Plant Mechanic	Logistics Analyst
Air Crew Officer	Airline Pilot, Copilot, and Flight Engineer	Logistics Specialist
Flight Attendant	Airman or Airwoman	Transportation Analyst
Air Marshal	Ambulance Driver	Import/Export Clerk
Air Traffic Controller	Boatswain (Bosun)	Dispatcher
Air Vice-Marshal	Bulldozer Operator	Warehouse Associate
Aircraft Body & Bonded Structure Repairer	School Bus Driver	Quality Control Clerk
Aircraft Cargo Handling Supervisor	Transit and Intercity Bus Driver	Traffic Office Specialist
Aircraft Engine Specialist	Bus, Truck and Diesel Engine Specialist	Railroad Worker
Aircraft Launch and Recovery Officer	Captain	Ship Engineer
Aircraft Launch and Recovery Specialist	Car and Wagon Driver	Logistician
Aircraft Mechanic and Service Technician	Chief Engineer	Captain (Maritime)
Aircraft Rigging Assembler	Chief Officer	Logistics Manager
Aircraft Structure Assembler, Precision	Commercial Diver	Aircraft Structure, Rigging, and Systems Assembler

## Conclusions

This study explored education reform aimed to examine the performance of the education system in Ghana. The selection of a course of study in a Senior High School in Ghana as the foundation to a student’s career aspiration has always been a challenge for students, parents, guardians and the school authorities due to the policy of limited number of the main Courses Offered by the schools. Students attend school from basic to secondary level for an average of 12 years, only to discover that they could have learn the same content in 6 years of half capacity, compared to their counterpart in other countries. Ghana’s education system is rated poor due to courses and curricula content, which resulted in waste of human capacity resources.

The key findings revealed urgent need of education reform focused on courses and curricula development and capital investment in education. The significance of Common Career Selective Programme (CCSP) offers the solution to the challenges enumerated. Adoption and implementation of CCSP by the key policy makers, and to provide funding, infrastructure and the needed resources. To improve the quality of education, the actionable recommendations outline an itemized measures required to solving the challenges by translating human capital index into sustainable national development.

## Recommendations

• Domestic and Regional stakeholders and collaborators to reform the current educational system, develop the necessary curriculums for adoption and implementation CCSP for EFA.
• Model schools equipped with the necessary facilities and resources to experimentally implementation CCSP for EFA as an action to drive development framework of EFA by 2030.
• Facilitator for CCSP for EFA to begin modernization of teaching and learning to enhance their knowledge and skills using Competent Base Training (CBT).
• Method of teaching for CCSP for EFA could be based on 80% for all Academic work and 20% hands-on or internship programmes, and 80% practical’s and 20% theory for all trade and apprentice programmes.

## Data availability

The data used for the study was based on electronic copy of Ministry of Education, Ghana Education Service, Ghana Senior High Schools Annual Digest 2019/2020 ( https://ges.gov.gh) Annual-Digest-2020-ecopy.pdf. The number of Courses Offered and School Statistics were tabulated from the “Annual Digest 2019/2020”.

The data tabulated from “2021 Second Cycle Schools Register” for the Academic Year 2019/2020, where the numbers of Courses Offered and the Students Statistics of the number of students in (SHS 1), (SHS 2), and (SHS 3).

## Acknowledgements

Ministry of Education, Ghana Education Service, Ghana Senior High Schools Annual Digest 2019/2020. ( www.ghanaschooinfo.org|© 2020.)

Ministry of Education, Ghana Education Service, 2021 Second Cycle Schools Register

The *World Book Encyclopedia*, 2001, “Career”. Duane Brown, “Career” *The World Book Encyclopedia*, 2001, Vol. 3, pp 213. And David A. Jepsen, “Career Education” *The World Book Encyclopedia*, 2001, Vol. 3, pp 214-235.
