# Supplementary material for: Education Reform using Common Career Selective Programme (CCSP) to Promote Education-for-All (EFA)
Source: F1000Res. 2025 Oct 29;14:679. Originally published 2025 Jul 9. [Version 2] doi: 10.12688/f1000research.163862.2 (PMC12739364; doi:10.12688/f1000research.163862.2)
Supplement: Peer review history

# Reviewer report: Reviewer response for version 2

**DOI:** 10.5256/f1000research.189281.r439792

**Published:** 2025-12-31

**Authors:** Hamid Asghari

**Affiliations:** 1 Karlstad University, Karlstad, Sweden

The paper addresses challenges in Ghana’s education system, including poor quality and limited course offerings, and proposes the Common Career Selective Programme (CCSP) as a reform strategy. CCSP expands the current seven courses into 18 career-oriented tracks to enhance inclusivity, equity, and relevance to labour market needs. The paper outlines seven actionable recommendations for policy integration, curriculum development, capacity building, and monitoring.

**Strengths**

 1) The topic is highly relevant and aligned with SDG 4 and Education-for-All goals.

 2) The paper provides practical recommendations for policy and curriculum reform.

 3) The attempt to link career guidance and curriculum design is commendable.

**Weaknesses and points for major revision**

 1) Methodology: The paper claims to use “content analysis” and “course and curricula modification”, but the process is unclear. There is no detailed description of steps, criteria, or how data informed the design of the 18 courses.

 2) Result transparency: The result is a list of 18 courses and policy recommendations, but it is not evident how these were derived from the data. The paper mixes normative policy ideas with scientific presentation, making it difficult to assess rigour.

 3) Research neutrality: The aim is value-laden (“to analyse gaps”), and headings such as “Poor education in Ghana” reinforce a negative framing. The paper lacks balance and does not acknowledge any strengths or progress in the system.

 4) Theory integration: Big Five and Holland’s RIASEC are mentioned but not applied in the analysis. The theoretical section feels disconnected from the results.

 5) Knowledge contribution: It is unclear what new insights the paper offers compared to existing reform discussions.

 6) International comparison: No reference to similar reforms in other countries, which would strengthen the argument.

 7) Ethical reflection: While equity is mentioned in recommendations, there is no deeper discussion of policy implications for marginalised groups.

 8) Structure and language: The paper is lengthy and repetitive. Policy recommendations should be clearly separated from the research analysis, and normative language should be toned down.

**Constructive suggestions**

 1) Clarify the methodology and explicitly link data to the proposed CCSP model.

 2) Neutralise the framing in the introduction and literature review; provide a balanced view of both challenges and strengths.

 3) Integrate theoretical frameworks into the analysis.

 4) Discuss the paper’s unique contribution and compare with similar reforms internationally.

 5) Add an ethical reflection on how CCSP affects marginalised groups.

 Restructure the paper to separate policy proposals from research content and reduce redundancy.

**Approval status**

 Approved with reservations (Major revision required) – The paper addresses an important issue and has potential, but significant improvements in methodology, neutrality, theoretical integration, and structure are necessary to make it scientifically sound.

Does the paper provide a comprehensive overview of the policy and the context of its implementation in a way which is accessible to a general reader?

Partly

Is the discussion on the implications clearly and accurately presented and does it cite the current literature?

Partly

Are the recommendations made clear, balanced, and justified on the basis of the presented arguments?

Yes

Reviewer Expertise:

Vocational Education and Training, Educational Policy, Curriculum Development, Teacher Education, Lifelong Learning, Educational Assessment, Comparative Education, Adult Education

I confirm that I have read this submission and believe that I have an appropriate level of expertise to confirm that it is of an acceptable scientific standard, however I have significant reservations, as outlined above.

# Reviewer report: Reviewer response for version 2

**DOI:** 10.5256/f1000research.189281.r438051

**Published:** 2025-12-24

**Authors:** Suhan WU

**Affiliations:** 1 Nanjing Polytechnic Institute, Nanjing, Jiangsu, China

**Peer Review Report**

**Title:** *Education Reform using Common Career Selective Programme (CCSP) to Promote Education-for-All (EFA)*

**Recommendation:** *Approved with Reservations — Major Revision Required*

**1. General Assessment**

 The manuscript addresses a relevant problem in Ghana's pre-tertiary education landscape and proposes the Common Career Selective Programme (CCSP) as a mechanism to improve course–career alignment. The aspiration of expanding educational pathways is commendable. However, the current version of the manuscript suffers from fundamental structural, methodological, and analytical limitations. Much of the text presents descriptive listings rather than scholarly argumentation, and the central claims are not supported by systematic data or evidence. Substantial revision is required to meet academic standards expected of a policy-oriented research article.

**2. Major Concerns**

**2.1 Lack of Evidence Supporting Key Claims**

 Several central assertions—such as " *The key findings revealed urgent need of education reform…"* (p.25) and the claim that Ghanaian students "learn at half capacity"—are not supported by empirical analysis within the study. While the authors cite the 2018 World Bank Human Capital Index, no methodological steps show how this data informs the CCSP model. No pilot study, comparative analysis, or evaluation data are presented to justify expanding the curriculum from 7 to 18 major courses.

**2.2 Absence of a Research Methodology**

 Although the manuscript states that content analysis was used, the methodology section does not describe:

- data sources selection criteria,
- coding procedures,
- analysis process,
- reliability or validity measures,
- how content analysis led to the derivation of 18 major courses.

As written, the methodology describes a policy design—but not a research method.

**2.3 Structural and Logical Inconsistencies**

- The *Results and Discussion* section appears in the middle of the paper (p.7), but the manuscript continues with lengthy reform proposals afterward (p.9–24), breaking the expected academic structure.
- The "six-step model" reproduced in the Results section simply reiterates a general curriculum development framework earlier cited (Kern's model), not findings derived from data analysis.

**2.4 Excessive Descriptive Listing without Analysis**

 More than half of the manuscript consists of long tables enumerating hundreds of possible careers for each proposed major. These listings:

- do not demonstrate relevance to curriculum reform,
- do not inform research questions,
- and do not contribute to policy argumentation.

The paper would benefit from substantial condensation and replacement of such lists with analytical content.

**2.5 Weak Linkage Between Theoretical Framework and Reform Proposal**

 The manuscript briefly references the Big-Five Personality Theory and Holland’s RIASEC model (p.3–4), yet does not apply these theories to justify:

- how the 18 major courses were conceptualized,
- how student interests and traits map onto the proposed structure,
- or how CCSP aligns with established educational or psychological theory.

The theoretical elements remain disconnected from the reform proposal.

**2.6 Recommendations Are Idealistic Without Feasibility Analysis**

 Actionable recommendations (p.7) call for national-scale policy integration, inter-ministerial coordination, and large-scale institutional changes. However, the manuscript provides no analysis of:

- resource requirements,
- implementation barriers,
- cost implications,
- teacher capacity,
- or political feasibility.

The recommendations therefore lack grounding in realistic policy analysis.

**3. Minor Concerns**

- Introduction is overly brief and should better contextualize global education reform trends and Ghana's policy landscape.
- Literature review is thematically inconsistent and requires reorganization to present a coherent conceptual flow.
- Several sentences are repetitive or reappear verbatim across sections, reducing clarity.
- Table 1 is duplicated in narrative form within the methodology and should be summarized analytically instead.

**4. Recommendations for Improvement**

 To strengthen the manuscript, I recommend:

1. **Substantially revising the structure** following conventional academic organization (Introduction → Literature Review → Methodology → Results → Discussion → Conclusion).
2. **Clarifying the research methodology**, including data sources, analytical procedures, and justification for the 18-course framework.
3. **Replacing extensive career listings** with analytical discussion of why curricular expansion is necessary and how it aligns with Ghana's needs.
4. **Integrating theoretical models** (RIASEC, Big-Five) into the rationale for course redesign.
5. **Providing evidence-based justification** for the CCSP model, including case studies, comparative policy insights, or pilot implementation data.
6. **Aligning the conclusion** with the actual analysis presented, avoiding overstated claims unsupported by data.

**5. Final Verdict**

 The manuscript requires **major revision** before it can be considered scientifically sound. With a clearer methodological foundation, deeper analysis, and tighter structure, the study could potentially contribute valuable insights into curriculum reform in Ghana.

**Recommendation: Approved with Reservations (Major Revision Required).**

Does the paper provide a comprehensive overview of the policy and the context of its implementation in a way which is accessible to a general reader?

Partly

Is the discussion on the implications clearly and accurately presented and does it cite the current literature?

Partly

Are the recommendations made clear, balanced, and justified on the basis of the presented arguments?

Yes

Reviewer Expertise:

Higher education；Supply chain management；Game theory；Chinese poetry and literature

I confirm that I have read this submission and believe that I have an appropriate level of expertise to confirm that it is of an acceptable scientific standard, however I have significant reservations, as outlined above.

# Reviewer report: Reviewer response for version 1

**DOI:** 10.5256/f1000research.180279.r409282

**Published:** 2025-09-12

**Authors:** Muhammad Khairul Rijal

**Affiliations:** 1 State Islamic University of Samarinda, Samarinda, Indonesia

The paper provides a clear and comprehensive overview of the proposed policy and the challenges it aims to address. The context of its implementation is also well-explained, with detailed descriptions of the current educational landscape in Ghana. the paper provides a clear and comprehensive overview of the proposed policy and the challenges it aims to address. The context of its implementation is also well-explained, with detailed descriptions of the current educational landscape in Ghana. While the paper cites key literature, it could benefit from a more thorough discussion of recent research specifically on educational reforms similar to the CCSP in other countries or regions. Drawing on examples from other African nations or globally could provide stronger evidence for the proposed changes. Additionally, more citations from recent studies on the impacts of education reforms would strengthen the credibility of the paper. To enhance the scientific rigor and relevance of the paper, the authors should address the following:

 1. Incorporate more empirical evidence and case studies on similar reforms from other regions.

 2. Provide a more detailed analysis of potential challenges and strategies for overcoming them.

 3. Clarify the roles of each stakeholder group in the implementation process.

 4. Propose a more detailed framework for evaluating the policy’s success and continuous improvement.

 5. Address the potential impact on marginalized groups to ensure inclusivity.

 These improvements will make the article more comprehensive and scientifically sound, enhancing its contribution to the field of educational policy.

Does the paper provide a comprehensive overview of the policy and the context of its implementation in a way which is accessible to a general reader?

Yes

Is the discussion on the implications clearly and accurately presented and does it cite the current literature?

Partly

Are the recommendations made clear, balanced, and justified on the basis of the presented arguments?

Yes

Reviewer Expertise:

Curriculum, education, teaching and learning

I confirm that I have read this submission and believe that I have an appropriate level of expertise to confirm that it is of an acceptable scientific standard, however I have significant reservations, as outlined above.

# Reviewer report: Reviewer response for version 1

**DOI:** 10.5256/f1000research.180279.r409273

**Published:** 2025-09-12

**Authors:** Asante George

**Affiliations:** 1 University of Cape Coast, Cape Coast,, Ghana

Topic:

 The topic is suitable for the work.

 Abstract:

 The general issue, together with the core concern of the study, is touched on. 

  Kindly do the following:

 1. Add the methodological component of the work in the abstract (design, data analysis).

 Introduction:

 Kindly link the 7th and 8th sentences well. There is a disjoint within. 

 Check your reference list well. The intext must be in line with the main reference list.

 Education Reform:

 Kindly link the 4th sentence (Asante et al, 2024) with the new educational reform of Ghana. 

 For instance: Ghana's new educational reform over the years...

 Kindly reframe the 1st sentence under 2nd paragraph.

 For instance: Ghana's education has experienced reforms over the years.

 Problem Selection of Courses offered:

 Please, limit redundancy. The introduction under this heading is repeated as the 1st bullet under ***Table 1***. Please, merge them.

 The research objectives are clearly touched upon. The objectives or work is very important in our contemporary world. It looks at how educational practices are preparing students to acquire the relevant skills, knowledge, and attitudes required at the workplaces, due to global mega-trends. The objectives again, respect the UN SDGs for development: The Era of Leaving No Student Behind.

 Literature source for the study is current.

 Policy Outcomes and Implications:

 Delete the 1st part. It is already stated as the objectives of the study: the last paragraph under "Problem Statement of Courses Offered".

 Merge the last paragraph under "Policy Outcome and Implications"  with the statement of the problem. 

 Follow it then with the research objectives.

 NB:

 Consider some theoretical underpinnings for the study, such as

 1. The Big Five Personality Theory (Goldberg, 1993)

 2. John Holland's Theory of Career Choice (RAISEC).

 Methodology:

 1. Consider the methodological component again.

 This is not a qualitative study but rather a content analysis write-up.

 Touch on the sources of data and how it was done (manually or electronically).

 Justify your usage of content review.

 Speak about data analysis (content review, comparative analysis, and thematic analysis).

 Justify your usage of these analyses.

 NB:

 Organization:

 Please consider reordering the work to make it simpler and clearer.

 1. Introduction

 2. Literature Review (Educational Reforms, Problem-Selection of Courses Offered, Actionable Recommendation, Theoretical Review).

 3. Methodology

    a. Research Design (Content Analysis)

    b. Data Analysis Method (Content Analysis, Comparative Analysis, Thematic Analysis).

 4. Results and Discussions

     a. Summary of key findings or results (This will be a summary of the literature. It can be in bullets.

      b. Discussions (here, elaborate a bit on the bulleted key findings. Please, let us hear your voice here.

 5. Conclusion and Recommendations.

Does the paper provide a comprehensive overview of the policy and the context of its implementation in a way which is accessible to a general reader?

Yes

Is the discussion on the implications clearly and accurately presented and does it cite the current literature?

Yes

Are the recommendations made clear, balanced, and justified on the basis of the presented arguments?

Yes

Reviewer Expertise:

Social Studies Education, Educational Assessment, Social Issues, Climate Change Education, SDGs for Education

I confirm that I have read this submission and believe that I have an appropriate level of expertise to confirm that it is of an acceptable scientific standard.
